# Exploring the chemical and pharmacological variability of *Lepidium meyenii*: a comprehensive review of the effects of maca

**DOI:** 10.3389/fphar.2024.1360422

**Published:** 2024-02-19

**Authors:** Norka Ulloa del Carpio, Diego Alvarado-Corella, Dante M. Quiñones-Laveriano, Andrea Araya-Sibaja, José Vega-Baudrit, Maria Monagas-Juan, Mirtha Navarro-Hoyos, Martha Villar-López

**Affiliations:** ^1^ Centro de Investigación Clínica de Medicina Complementaria—CICMEC, Gerencia de Medicina Complementaria, Seguro Social de Salud-EsSalud, Lima, Peru; ^2^ Bioactivity and Sustainable Development (BIODESS) Group, Department of Chemistry, University of Costa Rica (UCR), San Jose, Costa Rica; ^3^ Instituto de Investigaciones de Ciencias Biomédicas, Universidad Ricardo Palma, Lima, Peru; ^4^ Laboratorio Nacional de Nanotecnología, LANOTEC-CeNAT-CONARE, San José, Costa Rica; ^5^ United States Pharmacopeia (USP) Dietary Supplements and Herbal Medicines, Rockville, MD, United States; ^6^ Departamento de Medicina Preventiva y Salud Pública, Facultad de Medicina, Universidad Nacional Mayor de San Marcos, Lima, Peru

**Keywords:** *Lepidium meyenii*, maca, macamides, glucosilonates, macaenes, preclinical, clinical studies, pharmacology

## Abstract

Maca (*Lepidium meyenii*), a biennial herbaceous plant indigenous to the Andes Mountains, has a rich history of traditional use for its purported health benefits. Maca’s chemical composition varies due to ecotypes, growth conditions, and post-harvest processing, contributing to its intricate phytochemical profile, including, macamides, macaenes, and glucosinolates, among other components. This review provides an in-depth revision and analysis of Maca’s diverse bioactive metabolites, focusing on the pharmacological properties registered in pre-clinical and clinical studies. Maca is generally safe, with rare adverse effects, supported by preclinical studies revealing low toxicity and good human tolerance. Preclinical investigations highlight the benefits attributed to Maca compounds, including neuroprotection, anti-inflammatory properties, immunoregulation, and antioxidant effects. Maca has also shown potential for enhancing fertility, combating fatigue, and exhibiting potential antitumor properties. Maca’s versatility extends to metabolic regulation, gastrointestinal health, cardio protection, antihypertensive activity, photoprotection, muscle growth, hepatoprotection, proangiogenic effects, antithrombotic properties, and antiallergic activity. Clinical studies, primarily focused on sexual health, indicate improved sexual desire, erectile function, and subjective wellbeing in men. Maca also shows promise in alleviating menopausal symptoms in women and enhancing physical performance. Further research is essential to uncover the mechanisms and clinical applications of Maca’s unique bioactive metabolites, solidifying its place as a subject of growing scientific interest.

## Graphical Abstract

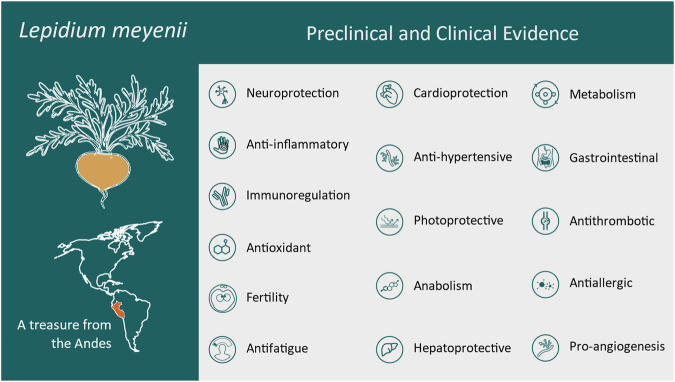

## 1 Introduction

*Lepidum meyenii* Walp, also known as maca, is a plant species belonging to the family Brassicaceae and native to the high Andean regions in Peru and Bolivia (Lim et al., 2015). Maca has been cultivated for more than two thousand years, with evidence of its cultivation observed in the central Peruvian Andes, specifically in Óndores district in Junín province (Gonzales, 2010; Gonzales and Alarcón-Yaquetto, 2018). For the Andean population, maca is considered a valuable commodity and its dried roots can be preserved for years (National Research Council, 1989). Its chemical composition is variable due to factors such as crop genetics, plant parts, growing conditions, crop management and analysis methods used (Wang and Zhu, 2019).

The first reports by Cobo and Ruiz in the mid and late 17th century, emphasized the fact that maca can grow in the coldest and wildest areas of the mountains, as well as its nutritional properties and ability to improve fertility, referred to by the natives (Ruiz, 1931; Cobo, 1956). At the end of the 17th century, Ruiz reported that women who could not conceive consumed this plant to treat their infertility; although this property has not been proven, he claimed that the consumption of maca in quantities acted as a stimulant (Ruiz, 1931). Later publications reported its use as a tonic for postmenopausal women and those trying to conceive (León, 1964), as a libido enhancer, but also as a cure for rheumatic pains and respiratory ailments, and as a laxative (Quirós and Aliaga Cárdenas, 1997). And contrary to the belief of the aphrodisiac effect of maca, a native source indicated that maca had never been consumed for such purposes, but instead as source of energy (Hermann and Bernet, 2009).

The numerous health benefits attributed to the consumption of maca have led to the consumption of dietary supplement products derived from maca. The Innova Markets Insights database reports 1,401 products in the global market containing maca on the label’s ingredient list (Innova, 2023), Although maca is considered a traditional food (or “not novel”) according to the regulations of many countries (e.g., Australia and New Zealand, United Kingdom, European Union) (Medsafe, 2006; Medicines and Healthcare Products Regulatory Agency, 2005; EU Novel Food Regulation 258/97, 2010), purified extracts from maca are not considered traditional foods. In 2009, the United States Pharmacopeia conducted an evaluation to identify potential serious risks to health or other public health concerns associated with the consumption of dietary ingredients derived from maca for their admission into the United States Pharmacopeia National Formulary (USP-NF) monograph development process (United States Pharmacopoeia, 2024). Ingredients evaluated during the USP-NF admission process included maca root, maca root powder and maca root dry extract (e.g., hydroalcoholic extracts). It was concluded that no serious adverse events were found for single-ingredient maca products. However, it was noted that the use of material not prepared following traditional methods of preparation (e.g., hydroalcoholic extracts) may contain components that would not normally be ingested when maca is eaten raw or prepared following traditional methods.

In 2017, the admission evaluation review was updated with relevant information, none of which suggested safety concerns. Currently, maca dietary ingredients comprise a wide range of ingredients including maca root powder, maca root gelatinized powder, and different types of maca root extracts purified to specific groups of bioactive compounds in maca (i.e., glucosinolates, macamides or aminoacids), the later ones requiring further evaluation for possible risk to health. Individual USP-NF quality monographs for these articles, including specifications, testing, and accepting criteria for the identity, composition, purity, and limits of contaminants, are currently in development.

The aim of this review is to explore the phytochemistry, pharmacological properties and clinical studies of *L. meyenii* Walp. providing a special emphasis on the detailed description of the effects derived from different types of maca dietary ingredients currently used in dietary supplement formulations in the global market. In addition to the efficacy studies in clinical or animal models, this review also intends to identify any potential serious risks to health or other public health concerns that could be associated with novel dietary ingredients, fractions of compounds, or individual compounds isolated from maca. This information could be used to update the current USP-NF admission evaluation report for maca.

## 2 Ethnobotany and ethnomedicine

Maca is considered a valuable commodity by the Andean population. Its dried roots can be preserved for years and are exchanged for staple foods in lower altitude communities, even reaching distant markets such as Lima. Both the dry, sweet and spicy root, and maca boiled in water, are considered delicacies. In Huancayo, Peru, maca pudding and maca jam are popular preparations (National Research Council, 1989). The cultivation of maca has evidence dating back more than 2000 years, between 700 and 600 BC, in the central Andes of Peru, specifically in San Blas district of Junín province, currently known as Óndores (Valerio and Gonzales, 2005). Although there are no written records due to the lack of writing in pre-Hispanic times, information about maca was transmitted orally between generations (Gonzales and Alarcón-Yaquetto, 2018), and some chroniclers and scientists, such as Cobo and Ruíz, collected data by entering the natural habitat of the plant (Gonzales, 2010).

As mentioned earlier, maca was attributed nutritional properties and fertility improving according to the accounts of the natives since the mid-seventeenth century (Ruiz, 1931; Cobo, 1956) After the Conquest, the Spaniards found that their cattle reproduced poorly on higher altitude lands, and the indigenous people recommended feeding them maca (National Research Council, 1989) and, as aforementioned, late in the 17th century, women consumed maca to treat their infertility according to Ruiz (1931) and León (1964).

However, not all native populations believed in its fertility-enhancing effect. Andean peoples consume an average of more than 100 g/day of maca root, where natives advise consuming only dehydrated maca root (Valerio and Gonzales, 2005) and boiled (Gonzales, 2012; Beharry and Heinrich, 2018) because of the belief that fresh maca can be harmful to health (Valerio and Gonzales, 2005).

Contrary to the aphrodisiac belief of maca, a native source indicated that it had never been consumed for that purpose, but for its energizing properties (Hermann and Bernet, 2009). Subsequently, other properties were attributed to it, such as improving memory and strengthening the immune system, although this may have been influenced by the hype on the internet and the massive promotion of maca during its boom in the 90s (Hermann and Bernet, 2009).

Reports about the properties and health benefits of maca have prompted studies. One of them, conducted more than 10 years ago in a population of the central Peruvian Andes, sought to determine if there was an association between maca consumption and the health status of people who consumed it in a traditional way. Maca consumption was found to be linked to better health, lower incidence of fractures, fewer signs and symptoms of mountain sickness, lower body mass index, and lower systolic blood pressure (Gonzales, 2010). Currently, several studies have described various beneficial properties of maca in health, among the most frequent are the effects on the male and female reproductive system, in the digestive system, in dermatological care, in neurological development, in metabolism; and as an energizer, antioxidant, neuroprotective, anticancer, hepatoprotective, antimicrobial, immunoregulatory, and photoprotective (Gonzales and Alarcón-Yaquetto, 2018; Wang and Zhu, 2019; Peres et al., 2020; Chen et al., 2021).

## 3 Phytochemistry

The chemical composition of maca varies due to factors such as crop genetics, plant parts, growing conditions, crop management, and the analysis methods used (Wang and Zhu, 2019). Dried hypocotyls contain between 13% and 16% protein, being rich in essential amino acids, while fresh hypocotyls contain more than 80% water, making them a low-calorie, nutrient-rich food (Valerio and Gonzales, 2005; Lim et al., 2015). In general, maca contains 10.2% protein, 59% hydrolysable carbohydrates, 2.2% lipids and 8.2% fiber. Dried hypocotyl also contains minerals such as iron, calcium, copper, zinc and potassium (Gonzales, 2012). Differences in mineral levels have been observed, whereas maca grown in China have higher concentrations of copper and sodium, while maca grown in Peru have higher concentrations of zinc (Zhang et al., 2015). In addition, differences in the mineral content of hypocotyls have been reported at different altitudes, with a higher concentration of phosphorus in the highlands and a higher concentration of iron in the lower areas (Vega, 2017).

As for the secondary metabolites, a significant amount of them have been identified, among the most notable: alkaloid compounds, such as lepedilins and macapirrolins (Chen et al., 2021; Todorova et al., 2021), thiazoles, macahidantoins, macaridine, alkaloids (Zhou et al., 2017a; Chen et al., 2021), macamides, macaínas (Zhou et al., 2017b; Chen et al., 2021; Todorova et al., 2021), glucosinolates (Zhou et al., 2017b; Gonzales and Alarcón-Yaquetto, 2018; Chen et al., 2021; Todorova et al., 2021), polysaccharides, polyphenols, sterols, free fatty acids, and flavonoids (Gonzales and Alarcón-Yaquetto, 2018; Chen et al., 2021; Todorova et al., 2021). In this regard, in a multivariate factorial study of 70 commercial samples of maca and raw tubers from China and Peru (Geng et al., 2020), differences were found between the secondary metabolites of the maca samples among these countries, as well as within the same country with respect to their ecotypes.

### 3.1 Macamides

**FIGURE 1:**
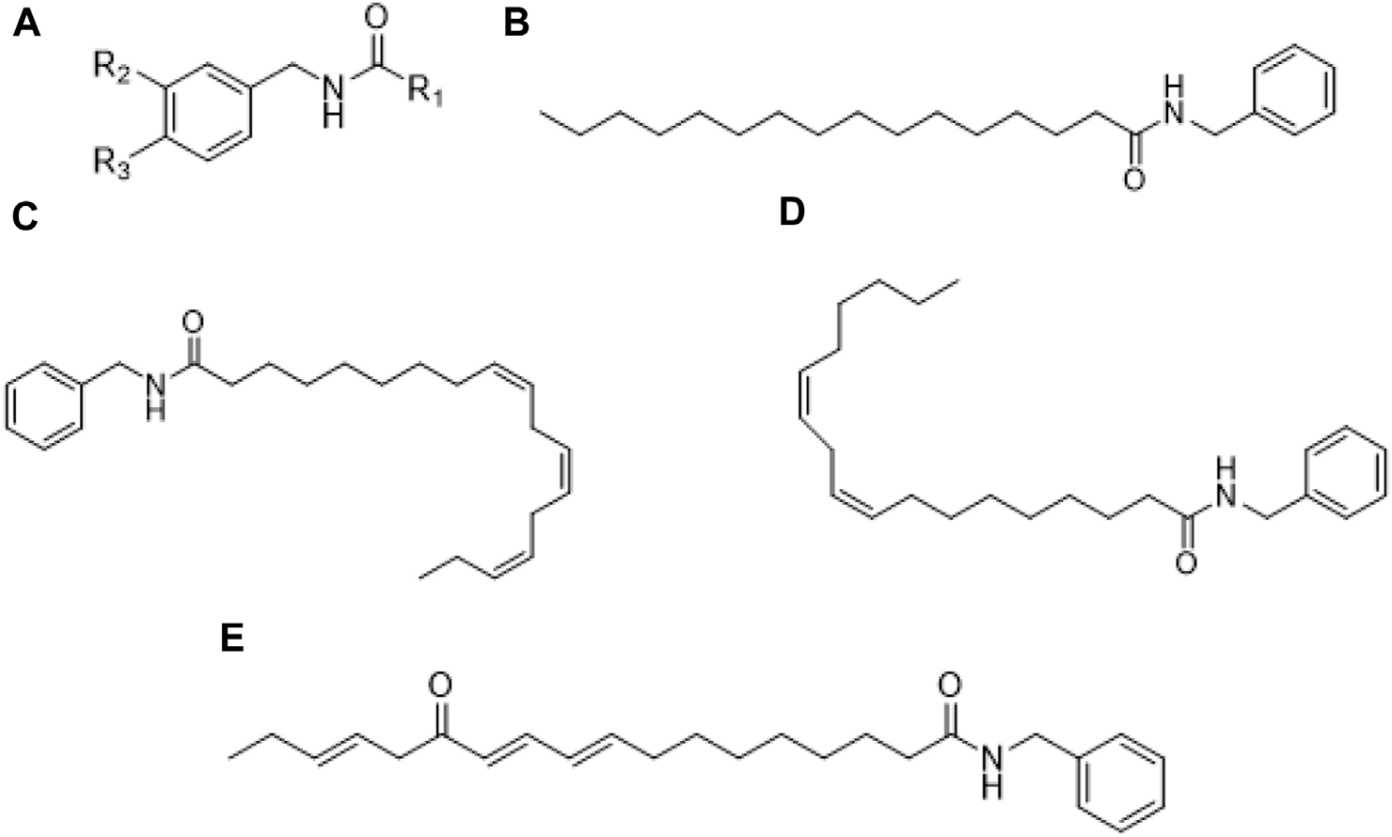
Chemical structure of macamides: **(A)** General structure; **(B)** N-benzyl-hexadecanamide; **(C)** N-benzyl-(9Z,12Z,15Z)-octadecatetraenamide; **(D)** N-benzyl-9Z,12Z-octadecadienamide; **(E)** N-benzyl-13-oxo-9E,11E,15E-octadecatrienamide.

Macamides are secondary and bioactive metabolites identified only in maca (Todorova et al., 2021), specifically in hypocotyls (Gonzales and Alarcón-Yaquetto, 2018). and are considered characteristic marker for quality assessment (Xia et al., 2021). Structurally, macamides are nonpolar N-benzylalcamides of fatty acids (Gonzales and Alarcón-Yaquetto, 2018), which vary with respect to hydrocarbon chain length and degree of unsaturation (Zhu et al., 2020). Its structural core (Figure 1) is constituted by a benzylamine or m-methoxybenzylamine moiety with alkyl groups of fatty acids such as oleic, linoleic, and linolenic attached through an amide bond (Zhu et al., 2020).

Within macamides, the biosynthesis pathway allows for a lot of diversity based on the fact they are derived from fatty acids or modified fatty acids (i.e., macaenes), and their isomers, resulting in N-benzyl-octadecadienamides and N-benzyl-octadecatrienamides*,* in addition to N-benzyl-oxo-octadecadienamides and N-benzyl-oxo-octadecatrienamides (Xia et al., 2021).

The effects of the drying process on macamide levels have been investigated. One study found that traditional (open-air) drying of maca hypocotyls in the Andes or industrial drying (flakes and ovens) causes hydrolysis of lipids and glucosinolates, and the release of significant amounts of unsaturated free fatty acids and benzylamines, precursors that correlate well with macamide biosynthesis (Esparza et al., 2015). Another study compared the concentration of macamides in air-dried and freeze-dried maca tubers. Macamide content was found to be higher in air-dried tubers, and new macamides were described (Xia et al., 2021).

### 3.2 Glucosinolates

**FIGURE 2:**
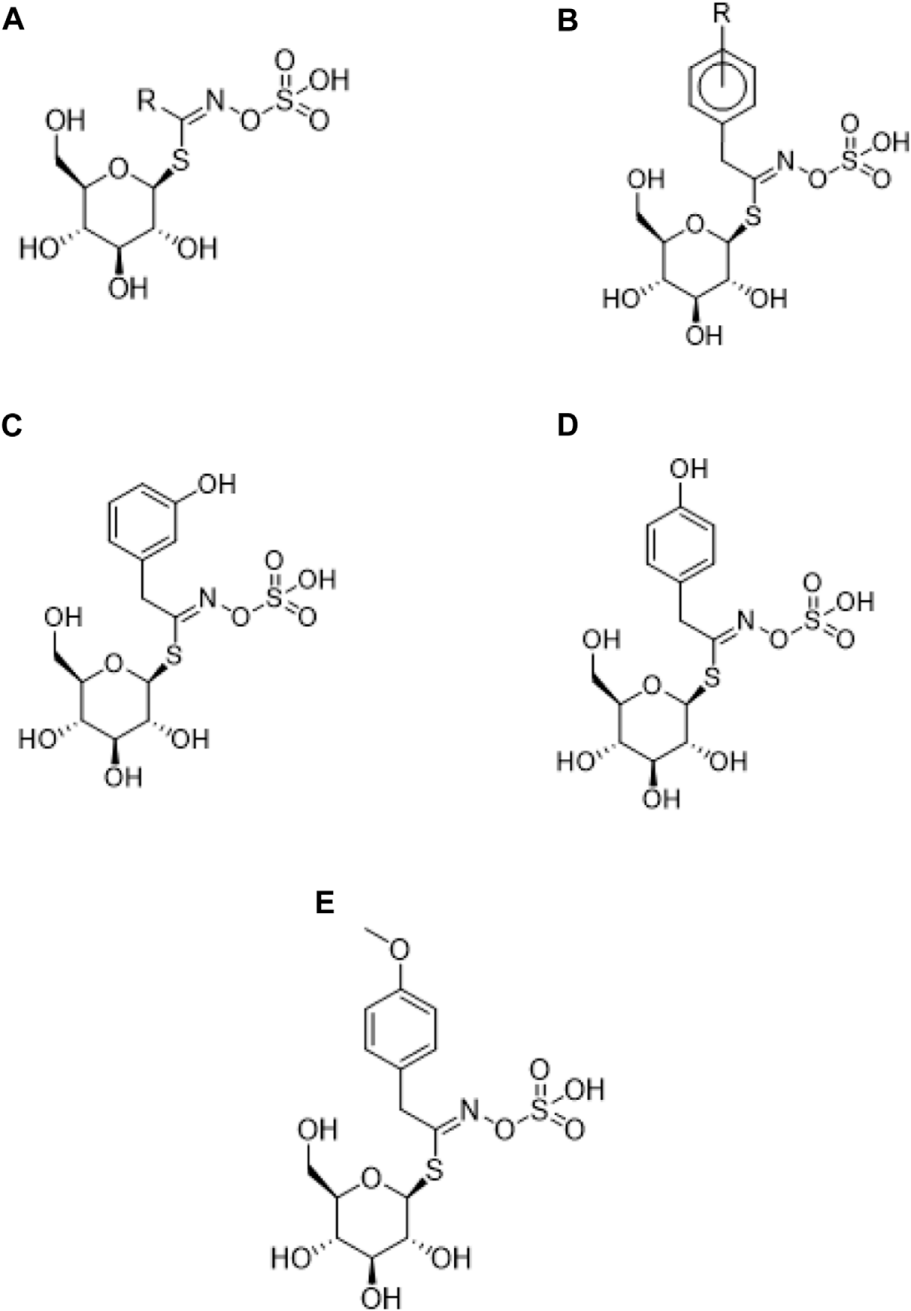
Chemical structure of glucosinolates. **(A)** General chemical structure; **(B)** Structure of Glucotropaeolin; **(C)** Glucolepigramin (3-hydroxybenzylglucosinolate); **(D)** Sinalbin (4-hydroxybenzylglucosinolate); **(E)** Glucolimnanthin (m-methoxybenzyl glucosinolate).

Glucosinolates are sulfur-rich hydrophilic anionic precursors found in plants of the Brassicaceae family (Chen et al., 2017b; Wang and Zhu, 2019), but are also present in 15 other dicotyledonous angiosperm families in which they are responsible for their pungent taste (Huang et al., 2018; Wang and Zhu, 2019). Within these families, approximately 120 glucosinolates have been identified, of which at least nine have been found in maca (Huang et al., 2018), with some of higher concentration and others in smaller quantities or in traces (Perez et al., 2021). Structurally, glucosinolates are N-hydrosulfates of β-thioglucoside (Huang et al., 2018; Perez et al., 2021). These compounds are formed by a β-D-glucopyranose attached to an ester (Z)-N-hydroxyminosulfate through a sulfur atom (Huang et al., 2018), and a variable side chain (R) derived from amino acids (Villanueva et al., 2019) as shown in Figure 2. The subclassification of glucosinolates is granted by the type of amino acid that constitutes their side chain. The amino acids alanine, leucine, isoleucine, methionine and valine form the side chain of aliphatic glucosinolates; phenylalanine and tyrosine are precursors of aromatic glucosinolates; and tryptophan of indole glucosinolates (Villanueva et al., 2019).

Benzylglucosinolates have been suggested as a chemical marker of maca bioactivity. However, this was ruled out as glucosinolates can be readily metabolized into isothiocyanates and these, in turn, into other metabolites (Gonzales, 2012); and they can be metabolized both in the plant and in the body (Gonzales and Alarcón-Yaquetto, 2018). In addition, benzylglucosinolates are also present in mashua (*Tropalum tuberosum*) (Gonzales, 2012).

The composition of glucosinolates in maca varies according to the stage of development, plant parts, cultivation practices, ecotype, pre- and post-harvest handling, and product type. A variation in glucosinolate content has been reported in seven different maca roots, ranging from 0.28 to 1.64 mg/g. Fresh roots have the highest total amount of glucosinolates, followed by seeds, shoots, dried roots, and fresh leaves (Wang and Zhu, 2019). A recent study reaffirmed the predominance of glucotropaeolin in the hypocotyls of maca (root powder); while, in the seeds, glucolepigramin/glucosinalbin (3/4-hydroxybenzylglucosinolate) was the most abundant. Such findings suggested an important role of the latter as a precursor in the biosynthetic pathways of other secondary metabolites, as this is the most abundant compound in maca seeds (Perez et al., 2021).

Other studies have reported a total glucosinolate concentration of 25.66 μMol/g (Lim et al., 2015); where aromatic-type glucosinolates make up 99% of the total, and are represented mostly by benzyl glucosinolate (glucotropaeolin Figure 2A.) with 16.94 μMol/g and p-methoxybenzyl glucosinolate (Figure 2E) with 6.38 μMol/g (Huang et al., 2018). It has been described that fresh maca hypocotyls contain up to 100 times more glucotropaeolin than other dicotyledonous angiosperms such as cabbage, cauliflower and broccoli; having been reported to correspond to approximately 1% of the fresh weight of maca (Wang and Zhu, 2019) and accounting for 80%–90% of its total glucosinolates (Villanueva et al., 2019; Wang and Zhu, 2019), followed by glucolimnantin (Huang et al., 2018).

### 3.3 Macaenes and free fatty acids

Macaenes are derived from long-chain unsaturated fatty acids and can also be found in other plants, such as mugwort leaves, eggplant calyx, and tomato fruits (Chinetti et al., 2000; Takahashi et al., 2011; Takahashi et al., 2014). Some of these long-chain unsaturated fatty acids have been found to behave as peroxisome proliferator-activated receptor (PPAR) agonists, which are ligand-activated transcription factors that can influence lipid metabolism. PPAR activation may increase fatty acid oxidation and reduce circulating and cellular lipid levels in obese diabetic individuals (Kim et al., 2011).

As mentioned above, authors have suggested that the formation of macamides in the postharvest drying process is related to macaenes and other long-chain fatty acid derivatives (Xia et al., 2019). It was then proposed that macamides are synthesized by the reaction of benzylamine or one of its substitutes and enzyme-catalyzed long-chain fatty acids or macaenes during the drying process (Xia et al., 2021). So far, more than 50 different compounds between fatty acids and macaenes have been identified in different studies on *L. meyenii* (Zheng et al., 2000; Muhammad et al., 2002; Esparza et al., 2015; Fu et al., 2021; Xia et al., 2021).

### 3.4 Biosynthesis

**FIGURE 3:**
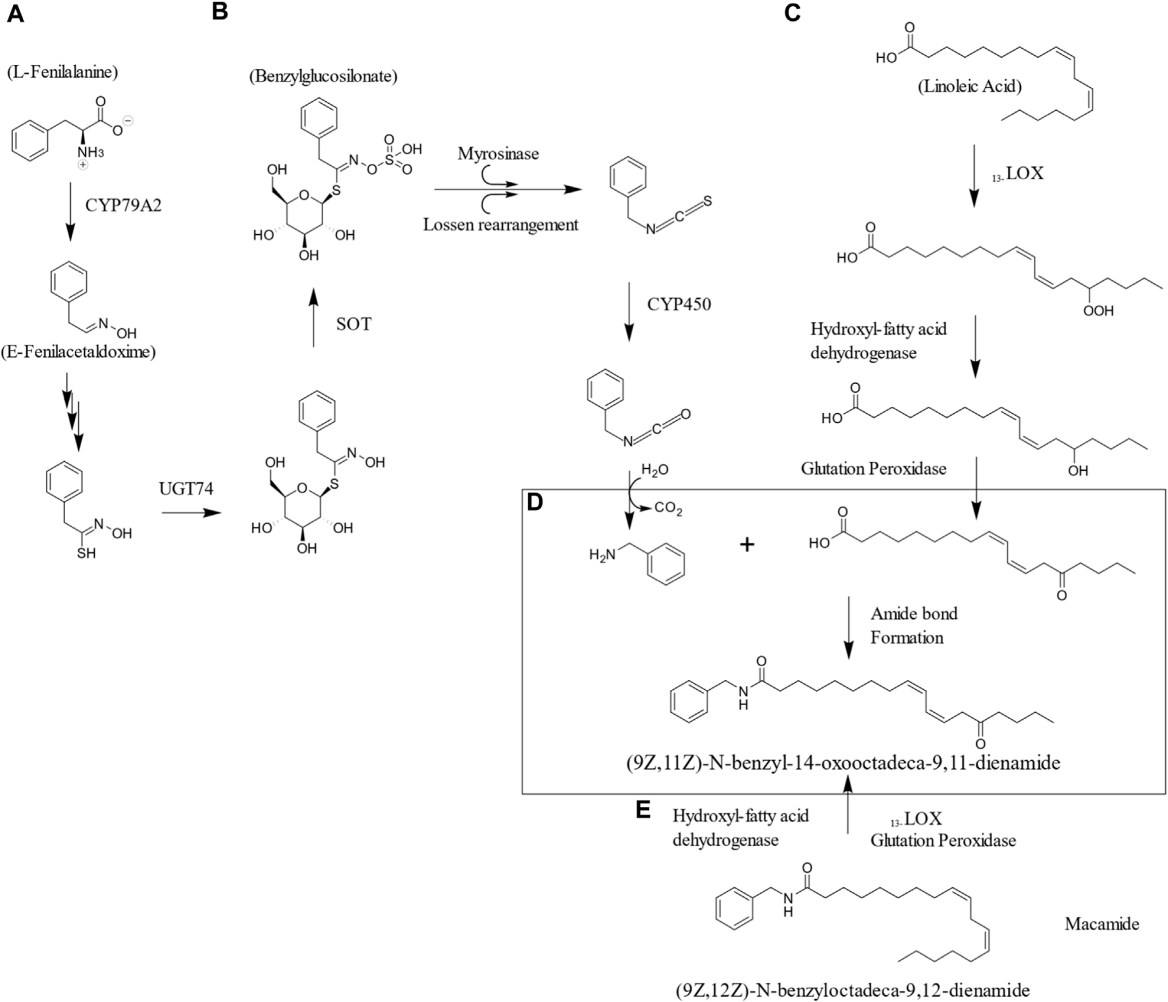
Hypothetical biosynthetic pathway to exemplify macamides formation during natural air-drying process. **(A)** The biosynthesis of benzyl glucosinolate from phenylalanine comprised the formation of (E)-Phenylacetaldoxime as intermediate produced by CYP79A2. This later transform into thiohydroximate and is glycosylated and sulfated. The unstable sulfated compound leads to isothiocyanate formation by the Sulfotransferase (SOT) and Glucosyltransferasa (UGT74) (Blažević et al., 2020). **(B)** The enzymolysis of the benzylglucosinolate the generation of benzylamine is accomplished by (Zhang et al., 2023). **(C)** In this example Linoleic acid serves as the scaffold for the formation of 9-hydroperoxy-10E,12Z-octadecadienoic acid and 13-hydroperoxy-9Z,12E-octadecadienoic acid through lipoxygenase enzymes, and then transfers the fundamental skeleton of macaenes using 9 or 13-hydroperoxy-10E,12Z-octadecadienoic acid. Glutathione peroxidase and hydroxy-fatty acid dehydrogenase enzymes catalyze the conversion of 9 or 13-hydroxy-10E,12Z-octadecadienoic acid into 9 or 13-oxo-10E,12Z-octadecadienoic acid. Isomerization also produces more isomers of octadecadienoic acid, resulting in the known chemical variety of macamides (Xia, 2021). **(D)** Once the hydrolysis of lipids and glucosinolates release significant amounts of unsaturated free fatty acids and benzylamines. These are precursors are the core for macamide biosynthesis (Esparza, 2015). **(E)** Isomerization can also occur once the amide bond is formed with the unaltered linoleic acid (Xia, 2021).

Macamides have been found to be the result of traditional post-harvest drying practices, as proposed in their biosynthesis mechanism, which involves the enzymatic degradation of glucosinolates to amines, the hydrolysis of storage and membrane lipids, and the formation of amide bonds (Esparza et al., 2015). In addition, it has been suggested the existence of factors during postharvest drying that may directly affect some events in the biosynthesis of macamides (Chen et al., 2017a). These factors may be present during the drying process, storage, cleaning, among others, the drying temperature, the shape of the tuber and the storage period being key factors for the accumulation of macamides (Xia et al., 2021). Figure 3 illustrates a possible biosynthetic pathway during natural air-drying process.

On the other hand, glucosinolate biosynthesis involves three main steps: elongation of the side chain (R) from aliphatic (methionine) or aromatic (phenylalanine) amino acids, nucleus formation with enzymes like S-glucosyltransferase and sulfotransferase incorporating glucose and sulfate, and modification of the side chain through oxidation and esterification (Villanueva et al., 2019). The catabolism of glucosinolates occurs through an enzyme known as myrosinase or thioglucosidase, which is stored in myrosin cells and is released in the presence of tissue damage (Huang et al., 2018), produced by some pathogenic infection or tissue disruption. This glucosinolate-myrosinase system hydrolyzes glucosinolates converting them into isothiocyanates, nitriles and thiocyanates, epithionitrils and oxazolidin-2-tiones (Perez et al., 2021). This system is also known as the mustard oil bomb (Huang et al., 2018; Perez et al., 2021) because isocyanates (mustard oils), the main product of hydrolysis, are potentially toxic to herbivorous insects (Huang et al., 2018); providing a chemical defense mechanism to the plant (Wang and Zhu, 2019; Perez et al., 2021). The initial increase in glucosinolates during drying can be associated with continued synthesis during the first postharvest week, when hypocotyls have not suffered sufficient tissue damage, explaining the initial presence of benzyl isothiocyanate in quantifiable amounts, but as time passes, other myrosinase hydrolysis products, such as benzylnitrile or benzylisocyanate, are the most predominant (Esparza et al., 2015).

### 3.5 Other secondary metabolites

Other secondary metabolites have also been identified, such as complex polysaccharides (Zhang et al., 2017a), imidazole (Cui et al., 2003; Jin et al., 2016) and pyrrole (Zhou et al., 2018) alkaloids, thiazoles, macahidantoins, macaridin, β-carboline (Chen et al., 2021), macaines (Chen et al., 2021; Todorova et al., 2021), sterols (Dini et al., 1994), free fatty acids and polyphenols (Gonzales and Alarcón-Yaquetto, 2018; Chen et al., 2021; Todorova et al., 2021), in addition to flavonoids including tricin derivatives (Bai et al., 2015).

### 3.6 Molecular pharmacology

The exact molecular mechanisms behind bioactivities of maca are still being elucidated. Maca has been recognized as an adaptogen used in recovery from illness, physical weakness, impaired mental function, and other conditions. Unlike synthetic adaptogens, the natural origin as plant extracts has an extremely rich phytochemical composition and physiological properties that are not due to a single one molecule, but to the combination of different compounds (Todorova et al., 2021). Early studies suggest that *L. meyenii* exerts hormonal balancing effect through Maca alkaloids, which act on the hypothalamus-pituitary adrenals (HPA) axis, but this hypothesis has not been confirmed (Muhammad et al., 2002). More recent evidence points to a probably synergetic mechanism for its effects on the HPA axis. An acute neuroendocrine response to adverse stress stimuli, as that counteract by adaptogens, is characterized by the tripartite activation of the three stress axes including the autonomic sympathetic nervous system, the direct neural innervation of the adrenal cortex, and a cascade of hypothalamic hormonal messengers (Spiers et al., 2015).

Maca extracts were found to alleviated mood stress and promoted hippocampal neurogenesis, which was attributed to increased 5-hydroxytryptamine (5-HT) and norepinephrine (NE) transmission through the endocannabinoid system (Gonzales-Arimborgo et al., 2016). Macamides are structurally similar to anandamide, and the lack of proportionality of concentration-dependent effects of both entities found in one study indicate that both might act by similar mechanisms in neuroblastoma cells, as it was demonstrated by a pentane extract of maca containing macamides (Pino-Figueroa et al., 2011).

Anti-fatigue effect of macamide N-benzyl-(9Z,12Z)-octadecadienamide in the hippocampus was evaluated, observing a decrease in iNOS and ROS levels, associated with an increase in the 5-HIAA/5-HT ratio and dopamine levels (Zhu et al., 2022b). Oral administration of maca extracts, maca powder and some isolated macamides has been shown to decrease levels of proinflammatory cytokines (TNF-α, IL-6, IL-1β) in hippocampus (Yu et al., 2020a; Yu et al., 2021), brain MDA (Olofinnade et al., 2021; Marín-Tello et al., 2021; Zhou et al., 2022 and AChE activity (Olofinnade et al., 2021); and increase levels of brain-derived neurotrophic factor (BDNF) (Yu et al., 2020a; Yu et al., 2021), serotonin receptors and hydroxyindoleacetic acid (5-HIAA) in hippocampus (Yu et al., 2021), glutathione peroxidase (GSH-Px) (Zhou et al., 2022), and neuronal density in hippocampal regions CA1 and CA3 (Yu et al., 2021). Recent data also suggest that the regulation of the gut–brain axis by maca may be one element of its therapeutic mechanisms (Hong et al., 2023). In this study, Maca extracellular vesicles exhibited antidepressant effects in UCMS mice accompanied by alteration in depression-related fecal microbiota and enhanced gut 5-HT metabolism, as well as elevated serum 5-HT concentrations and regulation of BDNF/TrkB/AKT axis.

N-benzylamides have antioxidant activity thanks to their ability to donate electrons (Chain et al., 2014); and they can capture free radicals thanks to their donor hydrogen bonds through their hydrophobic and hydrophilic regions (Zhu et al., 2020). In fact, the maintenance of a balanced redox state is critical for normal cellular homeostatic function within the neuroendocrine system (Spiers et al., 2015). Macamides are not the only contribuiting to the antioxidant effect, since the glucosilonate glucotropaeolin protected cardiomyocytes from oxidative stress and cell death through a mechanism independent of H_2_S signaling (Harvey et al., 2024).

Glucosinolates possess antitumor, antioxidant and antifungal activity (Todorova et al., 2021). They are considered the main source of anticancer activity of maca (Chen et al., 2021) because together with their metabolites, they exhibit chemoprotective characteristics by acting as inducers of phase 2 enzymes with possible antiproliferative, apoptosis-promoting, and redox-regulatory activities (Huang et al., 2018). These effects have been of special interest against HeLa cancer cell lines (Perez et al., 2021) and are mainly attributed to aromatic isocyanates, one of the products of glucosinolate hydrolysis (Villanueva et al., 2019).

It has been proposed that macamides may act on the nervous system through their inhibitory effect on the degradation of endocannabinoids, by disrupting the activity of the FAAH enzyme. One study evaluated this property showing that the effect on FAAH was dependent on macamide concentrations and suggesting its probable irreversibility (Alasmari et al., 2019). Macamides have shown antioxidant activity as one of their best-known properties and great potential as therapeutic agents; however, studies investigating this activity in humans are limited (Todorova et al., 2021). Its ability to bind to the endocannabinoid CB1 receptor has also been observed, acting as analogs of anandamide and exhibiting neuroprotective activity (Alasmari et al., 2019).

An acute neuroendocrine response to adverse stress stimuli is initiated by neurosecretory neurons in the paraventricular nucleus (PVN) of the hypothalamus, which release both corticotropin-releasing hormone and arginine vasopressin into the portal circulation of the pituitary gland. These two factors synergistically act on pituitary corticotroph cells to stimulate the release of the pro-opiomelanocortin peptide fragment, adrenocorticotropic hormone, into the circulation (Spiers et al., 2015).

The modulation mechanism of the HPA axis by the serotoninergic pathway, as suggested by Bromek et al., 2021, involves the negative regulation of liver cytochrome P450 through a decrease in growth hormone and an increase in T4 concentration due to the general activation of the brain serotonergic system. Network Pharmacology and *in silico* docking revealed that part of the mechanism of N-Benzylhexadecanamide (NBH). This molecule directly binds and inhibit the CYP1A2 (Cytochrome P450 Family 1 Subfamily A Member 2) active center, which in turn reduces the production of 16α-hydroxydehydro epiandrosterone, preventing excessive consumption of the testosterone (T) precursor DHEA and making it available for T synthesis (Zang et al., 2023).

**FIGURE 4:**
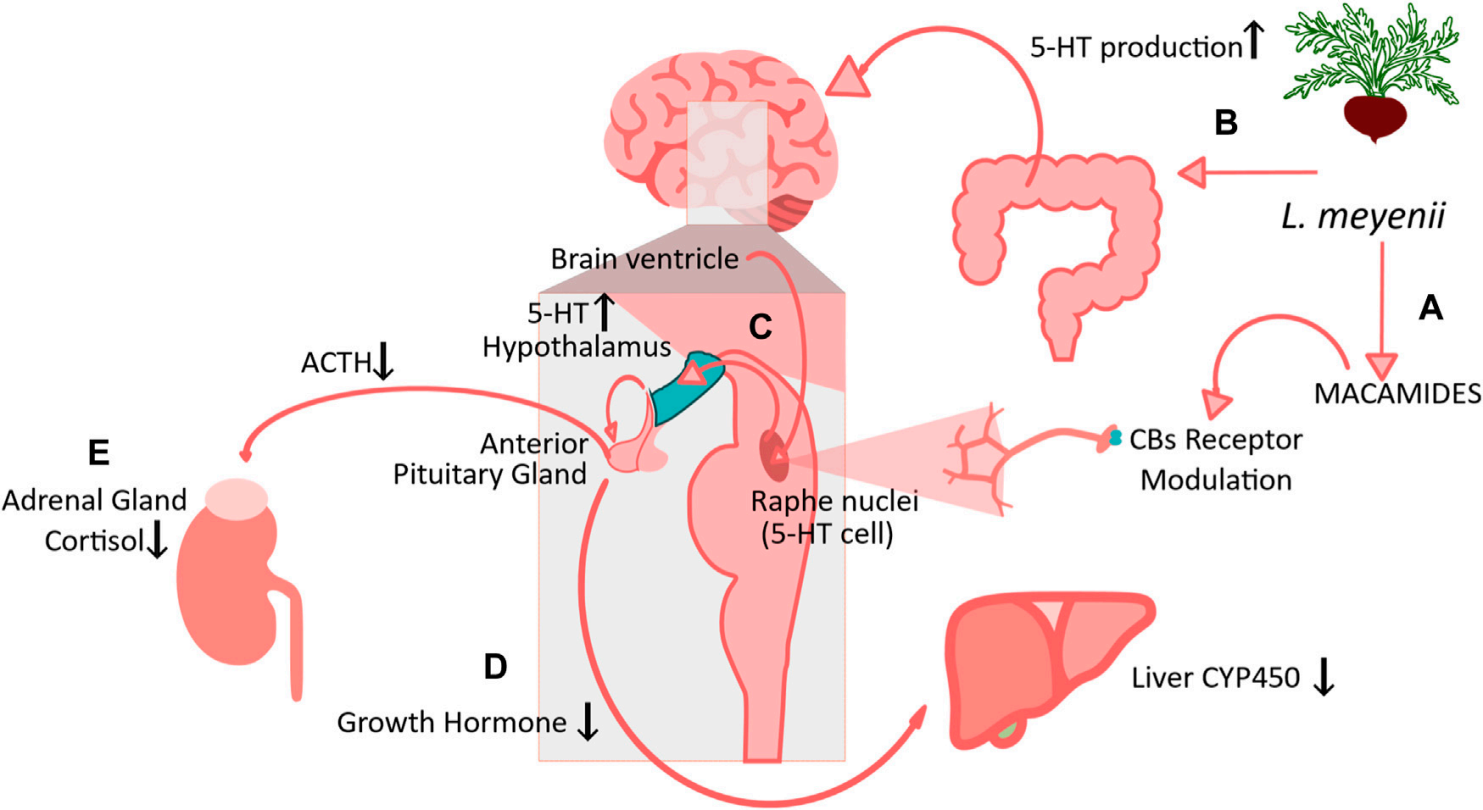
Maca possible mechanism of action based on recent evidence. **(A)** *L.* meyenii macamides can modulate HPA axis via serotoninergic pathwa*y* via CBs Receptors (Alasmari et al., 2019), but also **(B)** induce 5-HT production by microbiota and gut–brain axis modulation by maca (Hong et al., 2023) **(C)** Activation of 5-HT in the neurosecretory serotoninergic cells in the Raphe nuclei located in the paraventricular nucleus (PVN) of the hypothalamus which in turn reduce the anterior pituitary gland releasing Antherocotropina (ACTH) and of **(D)** growth hormone, which in turn reduce expression and activity of Liver CYP450. **(E)** The reduction of ACTH consecuently modulate a reduction in cortisol production in the adrenal glands (Meissner et al., 2006b).

Maca also acted as a toner of hormonal processes along the axis Hypothalamus-Pituitary-Ovaries, significantly stimulating production of Estradiol (E_2_) without significant effect on PRG. Simultaneously, the authors found suppression of blood follicle-stimulating hormone (FSH), luteinizing hormone (LH), triiodothyronine (T3), cortisol, and adrenocorticotropic hormone (ACTH) levels, along with an increase in blood Fe and bone density index. Additionally, alleviation of menopausal symptoms as per Kupperman Menopausal Index and Greene’s Menopausal Score, as well as a decrease in Body Mass Index (Meissner et al., 2006b). Figure 4 illustrates Maca possible mechanisms of action based on recent evidence discussed hereby.

## 4 Toxicology

Maca has a good safety profile (Brunetti et al., 2020), no fatal adverse effects have been reported (United States Pharmacopeia, 2012), and scientific research has found its consumption very safe (Beharry and Heinrich, 2018). Preclinical studies (summarized in Supplementary Table S1 in Supplementary Material) have reported its low *toxicity in vitro*, for instance maximum concentrations that preserve cell viability, such as 10 mg/mL for methanolic and aqueous extracts (Valentová et al., 2006), 500 μg/mL for red maca ethanolic extract (Chen et al., 2019), and 1 mg/mL for lyophilized black maca extract (Zhu et al., 2021). On the other hand, a wider variety of preclinical studies have reported *in vivo toxicity,* with the oral route of administration being the most evaluated, indicating a low level of acute oral toxicity in animals. For instance, pregelatinized maca has shown an LD50 greater than 7.5 g (Meissner et al., 2006a) and 15 g/kg/day in mice, and greater than 5 g/kg/day in rats (Meissner et al., 2006b), and its administration was safe up to 90 days of treatment (Meissner et al., 2006a).

It has been reported that maca’s aqueous extract showed no evidence of acute neurological toxicity at 10 g/kg/day (Tenci et al., 2017), nor liver toxicity at 1 g/kg/day (Gasco et al., 2007). Likewise, ethanolic extract did not show acute or chronic toxicity, suggesting an LD_50_ greater than 2 g/kg/day (Yu et al., 2020a). However, administration of 1.2 g/kg/day of maca powder for 30 days showed a significant elevation in serum urea nitrogen (BUN) levels (Li et al., 2017a). Regarding the intraperitoneal route, the administration of 60 mg/kg of a compound synthesized from maca (Macamide B) did not show acute toxicity (Yang et al., 2022); and another study that evaluated the teratogenic effect of 1 g/kg/day of lyophilized aqueous extract also showed no alterations in embryonic development (D´Arrigo et al., 2004).

Studies on maca toxicity in humans are limited (Supplementary Table S1 in Supplementary Material) and have mostly evaluated the oral administration of maca powder capsules for 12 weeks, where 2 g (Alcalde et al., 2020) to 3 g daily (Dording et al., 2008), as well as 3 g of maca extracts showed no serious adverse effects (Gonzales-Arimborgo et al., 2016). In addition, oral administration of 115 g/day of fresh micro-pulverized maca for 60 days did not result in liver or renal toxicity (Ronceros et al., 2005). Overall, good tolerance and no serious adverse effects were reported. However, two studies reported apparent adverse effects due to its consumption, one of vaginal bleeding in a 24-year-old woman (Srikugan et al., 2011) and another of a manic episode in a 27-year-old man with no psychiatric history (Quandt and Puga, 2016). While administration of 3 g of maca powder daily for 12 weeks presented some transient adverse events such as gastrointestinal upset, headache, and irritability (Dording et al., 2008).

Mild gastrointestinal disturbances were also reported with the administration of approximately 5 g of gelatinized maca daily for 30 days (Shin et al., 2023), while another study reported a moderate increase in a liver transaminase (AST) and diastolic blood pressure (DBP) with the administration of 0.6 g/kg/day of maca powder for 90 days (Valentová et al., 2008). Similarly, a study evaluating maca glucosinolates as part of an herbal supplement at doses of 3.3 g daily reported no adverse effects or immediate or late toxicity (Villar-López et al., 2021). On the other hand, an observational study conducted in 600 people from the Peruvian Andes reported that maca consumption was safe after evaluating some clinical parameters, liver and kidney function, and lipid profile, where no significant difference was observed between regular and non-habitual users (Gonzales, 2010).

Regarding the interaction of maca with other substances, there is very limited information. A recent review on the interaction of antidepressants with adaptogens reported a case of possible interaction between maca and a tetracyclic antidepressant (mianserin). It involved a 64-year-old male with other comorbidities such as prostate hyperplasia and arterial hypertension with heart failure, who was also taking medication for these conditions and experienced restless legs syndrome. The report suggested a possible mechanism involving the inhibitory effect of maca on a cytochrome oxidase (CYP3A4) involved in the metabolism of mianserin, leading to an increased concentration of mianserin in the blood and, consequently, the side effects of the mentioned drug (Siwek et al., 2023). However, when consuming multiple medications, it is not possible to conclusively determine a definite interaction.

## 5 Pharmacology

A total of 57 preclinical studies on maca were found reporting one or more effects, the most frequent being: neuroprotective, anti-inflammatory, immunoregulatory, antioxidant, anti-fatigue and on fertility. Other effects less frequently reported include: metabolic, gastrointestinal, cardioprotective, antihypertensive, photoprotective, anabolic, hepatoprotective, proangiogenic, antithrombotic and antiallergic.

### 5.1 Neuroprotective effects

The neuroprotective effects of maca were reported in 11 preclinical studies. *In vitro* studies have evaluated different maca formulations such as extracts (Rodríguez-Huamán et al., 2017; Yu et al., 2020a; Li et al., 2023), isolated compounds (macamides) (Gugnani et al., 2018), polysaccharide MP (Zhou et al., 2022) and glucosinolates (Tarabasz et al., 2022). Different neurotoxicity models have shown an increase in the viability of neuronal cell lines (Rodríguez-Huamán et al., 2017; Gugnani et al., 2018; Yu et al., 2020a; Zhou et al., 2022; Li et al., 2023) and a decrease in lactate dehydrogenase (LDH) (Rodríguez-Huamán et al., 2017; Yu et al., 2020a; Zhou et al., 2022), nitric oxide (NO) (Rodríguez-Huamán et al., 2017), intracellular calcium Ca^2+^, malondialdehyde (MDA), proinflammatory cytokines (COX-2, TNF-α, IL-6) (Yu et al., 2020a), reactive oxygen species (ROS) and cell cycle arrest (Zhou et al., 2022). Other studies have shown an increase in intracellular superoxide dismutase (SOD) (Rodríguez-Huamán et al., 2017), glutathione (GSH) (Gugnani et al., 2018), acetylcholinesterase (AChE) and butyrylcholinesterase (BuChE) inhibition (Tarabasz et al., 2022).

Regarding the possible mechanisms involved, ethanolic extracts have presented interaction with certain proteins (p65, IκBα) involved in the NF-κB pathway (Yu et al., 2020a), related to the response to cell damage. Within the isolated compounds, it has been suggested that macamides act through the cannabinoid receptor type 1 (CB1) due to their chemical structure similar to anandamide (endogenous cannabinoid) and by observing interaction with the nuclear receptor activated by peroxisomal proliferators gamma (PPARγ) (Gugnani et al., 2018), involved in cellular metabolism and homeostasis. As for the polysaccharide MP, it has been observed to inhibit the expression of certain proapoptotic proteins (p53, caspase 3 cleaved) (Zhou et al., 2022). Finally, an *in silico* study reported interaction between glucosinolates and tryptophan and histidine residues of AchE and BuChE enzymes, suggesting that they may act by blocking the active catalytic side of these enzymes (Tarabasz et al., 2022).

*In vivo* neuroprotective activity has been evaluated in different models of neurotoxicity: corticosterone (Yu et al., 2020a; Yu et al., 2021), diet rich in fats and sugars (HFHS) (Olofinnade et al., 2021), oophorectomy and/or radiofrequency radiation (RFR) (Marín-Tello et al., 2021), D-galactose (Zhou et al., 2022) and ischemic brain damage (Castro, 2022; Yang et al., 2022). Maca was used in the form of extracts (Yu et al., 2020a; Marín-Tello et al., 2021; Yu et al., 2021) and powder (Olofinnade et al., 2021) as well as in the form some isolated compounds, including macamides (Yu et al., 2021; Castro, 2022; Yang et al., 2022) and polysaccharide MP (Zhou et al., 2022).

Additionally, effects on the expression of cognitive and behavioral functions are reported, where oral administration of ethanolic extract and benzyloctadecathenamide reversed depressive behavior (Yu et al., 2020a; Yu et al., 2021), maca powder reversed memory impairment and anxious behaviors (Olofinnade et al., 2021), and lyophilized extract improved spatial memory (Marín-Tello et al., 2021). Likewise, parenteral administration of benzyloctadecanamide exhibited an improvement in post-ischemic damage neurobehavioral performance through recovery of somatosensory function (Yang et al., 2022) and decrease of sensory deficit (Castro, 2022). On the other hand, parenteral administration of benzyloctadecanamide in mice cerebral ischemic injury has been shown to reduce infarcted brain area (Castro, 2022; Yang et al., 2022) and cerebral edema (Yang et al., 2022).

Regarding the mechanisms involved, ethanolic extract has shown an increase in neurogenesis possibly associated with increased expression of the neuronal migration protein doublecortin, while benzyloctadecatrienamide has been shown to reverse the decrease in prosynaptic (synaptophysin, synapsin I and PSD95) and neurotrophic (p-CREB, BDNF) proteins associated with neurogenesis (Yu et al., 2021); while benzyloctadecanamide has been shown to participate in the PI3K/AKT prosurvival signaling pathway by increasing the expression of its proteins, and has been observed to potentiate autophagocytosis by regulating the expression of related proteins (Benclin 1, LC3B, p62) and inhibit neuronal apoptosis by regulating the expression of pro (p53, Bax, caspase, cleaved caspase-3) and antiapoptotic (Bcl-2) proteins (Yang et al., 2022).

The resemblance in the chemical structure of macamides to the neurotransmitter anandamide could theoretically suggest a potential addictive effect due to their interaction with endocannabinoid receptors (Alasmari et al., 2019), similar to certain psychoactive compounds found in cannabis. However, there are no studies that have reported such an effect, and there is not enough evidence to support such a claim; therefore, further research on this matter is still needed. Other components present in maca in minimal concentrations are the methyltetrahydro-β-carbolines, among which 1-methyl-1,2,3,4-tetrahydro-β-carboline-3-carboxylic acid (MTCA) has been suggested as an inhibitor of the MAO enzyme and a co-mutagen *in vitro*. Additionally, it has been proposed that compounds similar to MTCA may be associated with anxious behaviors, which are common in addictions (Piacente et al., 2002). At the moment, it is only possible to attribute the energizing or invigorating effect of certain maca components (macamides) to their interaction with endocannabinoid receptors as part of the various mechanisms proposed for the described effects, even suggesting a potential therapeutic effect on anxiety, depression, and pain (Zhu et al., 2020).

### 5.2 Anti-inflammatory and analgesic effects of maca

Anti-inflammatory and analgesic effects were reported in ten preclinical studies. *In vitro* studies were developed in human neutrophils (Purnomo et al., 2021) and different models of inflammation in macrophages (RAW 264.7) (Liu et al., 2021; Zou et al., 2021; Yan et al., 2022; Li et al., 2023) and enterocytes (Caco-2) (Chen et al., 2022). The anti-inflammatory activity of multiple fractions and components of maca ethanolic extract was evaluated, where the aqueous fractions of 75% methanol and n-hexane, and the compound macapirrolin A exhibited greater inhibition of superoxide anion and elastase (Purnomo et al., 2021). Among other compounds studied, lignan epipinoresinol and polysaccharides MC-1 and MC-2 were shown to inhibit pro-inflammatory factors such as IL-6 (82), TNF-α, IL-8 and INF-γ (Chen et al., 2022), and increased the anti-inflammatory factor IL-1 (Chen et al., 2022). In addition, a product of the hydrolysis of benzylglucosinolate (benzylisothiocyanate) and an extract of macamides presented considerable rates of nitric oxide inhibition (Yan et al., 2022; Li et al., 2023), and the former compound was able to overcome the effect of a nitric oxide synthase inhibitor (NOSI) (Yan et al., 2022).

*In vivo* studies in models of hepatitis (Zheng et al., 2018), pulmonary fibrosis (Ruiz Toledo et al., 2018) and benign prostatic hyperplasia (BPH) (Vásquez-Velásquez et al., 2020) applied various maca preparations orally. The lyophilized extract in hepatitis resulted in inhibition of serum and hepatic proinflammatory cytokines, inflammatory responses mediated by myeloid suppressor cells (MDSC), and expression of proapoptotic proteins (Zheng et al., 2018). On the other hand, the methanolic extract in pulmonary fibrosis was shown to decrease histological alterations, bronchoalveolar cell count and levels of pulmonary LDH and MDA, having a greater effect than prednisone in the latter marker (Ruiz Toledo et al., 2018). Finally, the hydroalcoholic extract in BPH showed a decrease in prostate stromal mass and area, and inflammatory cells (neutrophils, lymphocytes, mast cells); suggesting an inflammatory regulation promoted by the Th2 lymphocyte pathway due to the increase in anti-inflammatory cytokines (IL4, INF-γ) and decrease in a proinflammatory cytokine (TNF-α) (Vásquez-Velásquez et al., 2020).

The analgesic effect has been studied in different pain models such as: monoiodoacetate osteoarthritis (MIA), neuropathies due to chronic constriction of the sciatic nerve (CCI) and antineoplastic drugs (oxaliplatin and paclitaxel) (Tenci et al., 2017), and by LPS (Singh et al., 2020), where different maca preparations were administered orally. The aqueous extract decreased inflammatory pain, reflected by the decrease in joint hypersensitivity, with maximum effect at 15 min. Similarly, there was also a decrease in neuropathic pain, observed by the decrease in hyperalgesia in mechanical and pharmacological neuropathies, both with maximum effect at 30 min (Tenci et al., 2017). On the other hand, the administration of a macamide (benzyloctadecadienamide) showed greater inhibitory potency of soluble epoxide hydrolase (sEH) and better bioavailability than other macamides, as well as persistent antinociceptive effects for more than 6 h (Singh et al., 2020).

### 5.3 Immunoregulatory and antitumor effects of maca

Immunomodulatory and antitumor effects were evaluated in nine preclinical studies. *In vitro* studies in RAW 264.7 macrophage cell lines (Zhang et al., 2017b; Yang et al., 2021) and tumor lines of leukemia HL-60, lung cancer A549, liver cancer SMMC-7221, colon cancer SW480, and breast cancer MCF-7 (Fu et al., 2021; Liu et al., 2021; Yan et al., 2022) and 4T1 (Yang et al., 2021) evaluated the effect of compounds isolated from maca. Lepipyrroline A, macamides (benzyloctadechenamide and benzyloctadecadienamide) and a benzylglucosinolate hydrolysis product (BITC) showed a considerable cytotoxic effect on the aforementioned cell lines (Fu et al., 2021; Liu et al., 2021; Yan et al., 2022), which in some cases has come to match or exceed the effects of cisplatin, by administering lepipyrrolin A (Liu et al., 2021) or BITC (Yan et al., 2022), respectively. The MC-2 polysaccharide from maca induced differentiation to type 1 macrophages (M1), known for their proinflammatory activity, increasing the expression of proinflammatory markers (IL-6, iNOs). This polysaccharide was also shown to regulate the inflammatory response by converting type 2 macrophages (M2), known for their anti-inflammatory activity, into M1, partially decreasing the expression of anti-inflammatory markers (IL-10, Arg1) without completely inhibiting the proinflammatory effect described (Zhang et al., 2017a). Such regulation would prevent an excessive proinflammatory response, adequate enough to inhibit the growth of tumor cells, where it has been suggested that some macrophage membrane receptors (LR2, TLR4, CR3, MR) could be involved (Zhang et al., 2017b).

The effects observed with a maca polysaccharide (MP polysaccharide) were so promising that its use was proposed for a synergistic immunotherapy with chloroquine against a breast cancer cell line (4T1). This study also observed the immunoregulatory effects previously described in tumor-associated macrophages (TAMs), evidencing a decrease in the expression of anti-inflammatory proteins (CD206, Arg-1) and an increase in pro-inflammatory proteins (iNOS, TNF-α), associated with an increase in inflammatory cytokine transcription factors (TFEB, NF-κB p65). It was also observed that there was a modification of the cellular internalization and endocytosis pathways by transforming the MP polysaccharide into micelles (Yang et al., 2021).

*In vivo* studies in cyclophosphamide (CYP) immunosuppression models (Fei et al., 2020; Fei et al., 2022a; Alvarado et al., 2022) employed oral administration of maca extracts. Aqueous and polysaccharide extracts were shown to regulate the inflammatory response by increasing the expression of proinflammatory cytokines (IFN-γ, TNF-α, IL-2) and decreasing the expression of IL-4 (Fei et al., 2020; Fei et al., 2022a); while the hydroalcoholic extract showed an immunostimulatory effect on the humoral response to produce more post-sensitization antibodies (Alvarado et al., 2022). In studies with 4T1 breast cancer xenografts (Guo et al., 2020; Fu et al., 2021), intravenous administration of synergistic therapy with a maca polysaccharide (MPW) reduced systemic toxicity by doxorubicin and presented greater metastatic inhibition than standard treatment, being potentiated with cationic modification of the polysaccharide (C-MPW). It was also observed that it promoted the differentiation of TAMs causing the increase of pro-inflammatory factors (IL-12, TNF-α, INF-γ, iNOS) and the decrease of anti-inflammatory factors (IL-10, MMP9, VEGF) (Guo et al., 2020). On the other hand, the use of chloroquine associated with a polysaccharide in the form of amphiphilic micelles (MP-ss-PLGA) showed a greater inhibition of tumor growth and lung metastasis than the standard treatment, as well as an efficient intracellular supply of chloroquine and a greater antiangiogenic effect (Yang et al., 2021).

Evaluating possible mechanisms involved, it was found that aqueous and polysaccharide extracts maintained the balance between pro-(Th1) and anti-inflammatory (Th2) lymphocytes participating in the regulation of transcription factors for their differentiation by increasing the expression of T-bet (Fei et al., 2020; Fei et al., 2022a) and decreasing GATA-3 (Fei et al., 2020), and through NF-κB signaling pathways, STAT1 and STAT3 (95). The polysaccharide extract also increased the CD4^+^ lymphocyte count and, consequently, the CD4+/CD8+ ratio; in addition, it decreased lymphocyte apoptosis in the spleen by regulating the expression of pro factors (BAX, caspase-3) and antiapoptotic factors (BCL2) (Fei et al., 2022b).

### 5.4 Antioxidant effect of maca

The antioxidant effect was evaluated in six preclinical studies. *In vitro* studies were performed in direct trials (Fu et al., 2021; Ibrahim et al., 2022; Yan et al., 2022) and in models of oxidative damage in hepatocytes (HEp-2) (Zhang et al., 2017a) or macrophages (RAW 264.7) (Wang et al., 2018), with various maca preparations. Extracts of maca, macamides, macaenes and total glucosinolates had a capacity to capture free radicals (Fu et al., 2021; Ibrahim et al., 2022; Yan et al., 2022) and a reducing power (Fu et al., 2021; Yan et al., 2022) proportional to the dose (Yan et al., 2022). Methanolic extract and glucosinolates exhibited more potent antioxidant activity (Ibrahim et al., 2022; Yan et al., 2022); while macamides, crude extract and glucosinolates came to be compared with the antioxidant effect of ascorbic acid (Fu et al., 2021; Yan et al., 2022). In hepatocytes (HEp-2) with oxidative damage, the polysaccharide MP-1 showed an ability to scavenge free radicals (hydroxyl, DPPH, superoxide anion) and dose-proportional reducing power (Zhang et al., 2017a). Similar results were obtained in macrophages (RAW 264.7) with oxidative damage, where the ability to chelate iron and inhibit lipid peroxidation associated with increased cell viability was added; such effects were also reflected in the expression of some markers, decreasing the levels of ROS, MDA and LDH, and increasing the levels of GSH-Px, catalase and SOD (Wang et al., 2018).

The antioxidant effect *in vivo* was evaluated in models of hepatic oxidative damage by OH (Zhang et al., 2017b) and acrylamide (Ybañez-Julca et al., 2022), using the oral route. The aqueous extract was shown to decrease systemic levels of MDA (in erythrocytes, brain and liver), lipid peroxidation and liver transaminases (ALT, AST) (Ybañez-Julca et al., 2022). On the other hand, the polysaccharide MP-1 also showed similar effects, adding the decrease in serum levels of triglycerides, LDL cholesterol, γ-glutamyltranpeptidase, and the increase of antioxidant liver enzymes (SOD, GSH-Px, glutathione S-transferase), in addition to decreasing histopathological liver alterations (Zhang et al., 2017a).

### 5.5 Effect on fertility

This was one of the first claim about maca properties to be studied; ten recent preclinical studies were found, mostly aimed at male fertility. *In vitro* studies evaluated the effect of different maca extracts on cells of the male reproductive system, including Sertoli cells (Yoshida et al., 2018) and sperm (Leiva-Revilla et al., 2022; Prete et al., 2022). The hydroalcoholic extract increased testosterone production by Leidig cells and was involved in steroidogenesis; however, this steroidogenic effect declined with increasing age (Ybañez-Julca et al., 2022). The protective effect on sperm sample preservation was also evaluated, where maca extract in frozen sperm subjected to thawing showed a transient improvement in sperm motility, integrity and morphology, as well as an increase in acrosomal integrity and sperm vitality associated with a decrease in sperm DNA damage at 24 h (Leiva-Revilla et al., 2022). In another study, the use of aqueous extract in the preservation of sperm exposed to freezing evidenced an increase in sperm hyperactivation and the preservation of sperm DNA integrity at 24 h (Prete et al., 2022). Both studies agreed that these effects occur at low doses (Leiva-Revilla et al., 2022; Prete et al., 2022); while high doses (≥50 μL/mL) exhibited immediate harmful effects on seminal quality (Prete et al., 2022).

*In vivo* studies on male fertility in disease-free settings (Prete et al., 2018; Yoshida et al., 2018; Miranda et al., 2022) and in gonadal toxicity models induced by cyclophosphamide (Onaolapo et al., 2018), corticosterone (Yu et al., 2020a), environmental heat stress (Ragab et al., 2023) or busulfan (Zhou et al., 2023) employed oral administration of a variety of maca preparations: dry extract (Onaolapo et al., 2018; Miranda et al., 2022), hydroalcoholic extract (Yoshida et al., 2018), maca powder (Prete et al., 2018), ethanolic (Yu et al., 2020a; Zhou et al., 2023) and aqueous extracts (Ragab et al., 2023), and fractions of polysaccharides, oligosaccharides and small molecules (Zhou et al., 2023).

Under healthy conditions, dietary supplementation with maca powder increased sperm concentration and total sperm count after being exposed to refrigeration, improved some sperm quality parameters (total and progressive motility, acrosomal integrity), and decreased DNA fragmentation (Prete et al., 2018). On the other hand, the hydroalcoholic extract was shown to transiently increase serum testosterone levels in young male rats (Yoshida et al., 2018); and the dry extract reduced the thickness of the epididymis epithelium (tail) and increased testicular lymphatic sinuses (Miranda et al., 2022). The effect of maca in combination with *Tribulus terrestres* was also studied, resulting in an increase in testosterone levels, the diameter of the epididymal duct and lumen, and sperm concentration and percentage relationship of vessels (Miranda et al., 2022).

Under adverse conditions and gonadal toxicity, attenuation of oxidative stress was presented by decreasing MDA and increasing GSH, SOD, CAT (Onaolapo et al., 2018), GSH-Px and GST (Yu et al., 2020a). The dry, ethanolic, and aqueous extracts increased serum testosterone levels and some sperm quality parameters (concentration, motility) (Onaolapo et al., 2018; Ragab et al., 2023; Zhou et al., 2023). The ethanolic and aqueous extracts and benzyloctadecatrievide also decreased abnormal sperm concentration (Yu et al., 2020a; Ragab et al., 2023), and the aqueous extract increased sperm plasma membrane survival and functionality (Ragab et al., 2023). Histologically, ethanolic extract and benzyloctadecatrienamide preserved the morphology of seminiferous tubules (Yu et al., 2020a), as did the dry extract in the epididymis; the latter also showed an increase in the width of seminiferous tubules and the thickness of the germ cell layer (Onaolapo et al., 2018). The aforementioned macamide also reversed testicular toxicity, and ethanolic extract decreased spermatogenic tubule vacuolization and testicular lesions, eventually recovering spermatogenic epithelium and inhibiting testicular stromal proliferation (Yu et al., 2020b).

Effects on sperm production were also reported, where ethanolic extract and benzyloctadecatrienamide improved spermatogenesis by increasing primary spermatogonia and spermatocytes, and decreasing spermatogenic cell apoptosis (Yu et al., 2020b); even a decrease in latency to conceive and an increase in the number of products conceived was reported (Onaolapo et al., 2018). The possible mechanisms were attributed to the increased expression of PCNA and Ki67 in seminiferous tubules and to the regulation of proteins related to the Nrf2/ARE pathway, related to the intrinsic defense against oxidative stress (Jin et al., 2018).

Conversely, the *in vivo* effect on the female reproductive tract has not been as studied as its male counterpart. In one recent study the effect of aqueous extracts of two maca phenotypes (red and black) *versus* menopause induced in a high-altitude environment was evaluated. Partial uterotrophic protection close to estradiol was observed, evidenced by the preservation of uterine mass and the increase in cornified endometrial cells, without changes in the amplitude and frequency of contractions. In addition, a decrease in oxidative stress was evidenced by decreasing MDA (Ybañez-Julca et al., 2020).

### 5.6 Anti-fatigue effect

The anti-fatigue effect has been reported in seven preclinical studies. In *in vitro* studies using models of oxidative stress in skeletal muscle cells (C2C12) (Zhu et al., 2021; Zhu et al., 2022a) and lyophilized extract of maca, an inhibition of cell necrosis was observed (Zhu et al., 2021; Zhu et al., 2022b) with minimal compromise of viability (Zhu et al., 2021), evidencing a decrease in mitochondrial ROS levels (Zhu et al., 2021; Zhu et al., 2022a) and an increase in glycogen, ATP generation capacity, and mitochondrial membrane potential (Zhu et al., 2022a).

*In vivo* studies in forced swimming test (FST) fatigue models evaluated the oral administration of powdered maca preparations (Li et al., 2017a; Orhan et al., 2022), lyophilized extract (Zhu et al., 2021) as well as some isolated compounds such as polysaccharide A (Tang et al., 2019) and benzyloctadecadienamide (Zhu et al., 2022b). Maca powder decreased serum lactic acid (Li et al., 2017a; Orhan et al., 2022), serum MDA levels, and liver and muscle MDA, and increased muscle activity of the enzyme glutathione peroxidase (Orhan et al., 2022). There was also evidence of decreased levels of a proinflammatory transcription factor (NF-κB) and increased levels of some transcription coactivators involved in mitochondrial biogenesis (Nrf1, Nrf2, PGC-1α, SIRT1 and TFAM) (Orhan et al., 2022). Likewise, effects on physical endurance were evidenced by increasing swimming time (Li et al., 2017a; Orhan et al., 2022).

The lyophilized extract showed a decrease in the levels of LDH, LA, BUN and ROS, the latter at the serum and tissue level, and skeletal muscle damage associated with an increase in tissue redox coenzymes (NAD+, NADH) (Zhu et al., 2021). On the other hand, within the isolated compounds, polysaccharide A and benzyloctadecadienamide were shown to decrease serum LDH and BUN levels (Tang et al., 2019; Zhu et al., 2022b). Polysaccharide A also showed an increase in liver and muscle glycogen (Tang et al., 2019); while benzyloctadecanamide inhibited histopathological changes resulting from liver damage, decreased ROS and IL-1β levels, and positively regulated heme oxygen-1 (HO-1) expression.

Finally, the anti-fatigue effect of a mixture that included lyophilized maca extract was evaluated. Improvement in exercise capacity (Zhu et al., 2022c) and forelimb strength (Vásquez-Velásquez et al., 2020) was observed. The relief of fatigue was accompanied by a regulation of the intestinal flora, observing an increase in the amount of some beneficial bacteria such as *Lactobacillus* (Zhu et al., 2022a; Zhu et al., 2022c) and Akkermansi (Zhu et al., 2022a), associated with a decrease in some harmful bacteria such as *Candidatus planktophila* (Zhu et al., 2022a; Zhu et al., 2022c) and *Candidatus arthromitus* (Zhu et al., 2022a). Following these findings, the use of maca and other plants as prebiotics has been suggested (Zhu et al., 2022c).

### 5.7 Other effects

Other less studied effects were evaluated in 12 preclinical studies, including metabolic, gastrointestinal, cardioprotective, antihypertensive, photoprotective, anabolic, proangiogenic, antithrombotic, and antiallergic activity.

#### 5.7.1 Metabolic effect or on energy metabolism

An *in vitro* study evaluated the effect of ethanolic extract of maca on adipocytes and lung carcinoma cells (H1299). A biphasic effect, such as insulinomimetic and agonist, was observed, and an increase in glucose uptake in insulin-resistant cells was evidenced (H1299). This last finding suggested that maca does not act as a simple insulin receptor agonist, and the increase in glucose uptake could be mediated by more than one pathway. Likewise, AMP-activated protein kinase (AMPK) was suggested as a possible main mediator, with the activation of the insulin-Akt pathway secondary to that of AMPK (Chen et al., 2019).

*In vivo* studies on energy metabolism were performed in small mammals (mice, rats). In a context of immunosuppression, the aqueous extract showed an increase in body temperature, cAMP/cGMP index and the activity of the Na^+^/K^+^ and Ca^2+^/Mg^2+^ pumps. Similarly, an increase in levels of liver glycogen, LDH, leukocytes, platelets and hemoglobin were observed (Fei et al., 2020). Moreover, in the context of metabolic syndrome, the administration of two commercial presentations of maca powder exhibited regulatory metabolic effects. A decrease in weight gain and food intake was observed, associated with the decrease in hyperglycemia, dyslipidemia and oxidative stress (SOD), and the regulation of inflammatory markers (TNF-α, IL-10) (Olofinnade et al., 2021). The above effects may be related to an increase in leptin levels in adipose tissue and in the hepatic activity of insulin receptor substrate 1 (IRS1) and sirtuin 1 (SIRT1); effects reflecting increased satiety and decreased insulin resistance, respectively (Gencoglu, 2020).

#### 5.7.2 Gastrointestinal effect

*In vivo* studies in small mammals (rats, mice) with impaired gastric motility by atropine (Jin et al., 2018) and impaired nutrient digestibility by high-fat diet (Sahin et al., 2021) employed oral administration of two maca preparations. The administration of lyophilized aerial parts showed a decrease in gastric residue and an increase in intestinal propulsion; exhibiting an improvement in gastric emptying and intestinal peristalsis, respectively. An increase in serum levels of some prokinetic gastric hormones (MLT, GAS) was also observed (Jin et al., 2018). On the other hand, the administration of a commercial powder presentation increased the genetic expression of multiple nutrient transporters in jejunum and ileum, such as some peptide transporters (Pept1, Pept2), fatty acid (Fatp1) and glucose (Glut1, Glut2, Sglt1); suggesting an improvement in nutrient digestibility (Sahin et al., 2021).

#### 5.7.3 Cardioprotective effect

An *in vivo* study in rats with myocardial stunning due to ischemia/reperfusion using oral administration of a commercial form of maca powder reported significant cardioprotection independent of sex, but not at advanced ages. This effect was evidenced by increasing contractile and energetic recovery after ischemic event; a synergistic effect against mitochondrial dysfunction was also observed, including activation of NO-synthases, mKATP channels, and Na^+^/Ca^2+^ mitochondrial exchanger pathways (Colareda et al., 2021).

#### 5.7.4 Antihypertensive effect

An *in vitro* and *in silico* study with various extracts of maca root (methanolic, aqueous, and 50% methanoldichloromethane extracts) and some isolated compounds (glucotropaeoline, β-carboline alkaloids, succinic acid, 2,4-dihydroxy-3,5-cyclopenctyldieneic acid, macamides) evaluated the possible antihypertensive effect. In the *in vitro* tests, the methanolic extract showed the greatest inhibition of angiotensin-converting enzyme (ACE) and renin. On the other hand, the *in silico* analysis exposed the possible coupling mechanisms; compounds glucotropaeolina, β-carboline alkaloids, succinic acid and 2,4-dihydroxy-3,5-cyclopentildienoic acid showed greater affinity for the active site of ACE, whereas macamides showed greater affinity for the active site of renin (Ibrahim et al., 2022; Harvey et al., 2024).

#### 5.7.5 Photoprotective effect

The climatic conditions in which maca plants usually develops leads to a potential photoprotective effect, which was evaluated *in vivo* in mice exposed to irradiation with ultraviolet B (UV-B) rays. This study reported that the topical administration of a cream containing lyophilized aqueous extract at 5% and 15% inhibited epidermal thickening, presenting a photoprotection comparable to a commercial sunscreen with SPF 30. It was even observed that the epidermal thickness measurements treated with 15% aqueous extract were similar to those that were not exposed to UV-B radiation (Castañeda-Alarcón et al., 2021).

#### 5.7.6 Anabolic effect

An *in vitro* study evaluated the effect of maca extract on muscle growth, evidencing a stimulation of growth, differentiation and cell maturation through certain parameters: greater diameter and area of myotubes, higher differentiation index and greater multinucleation. Likewise, possible mechanisms involved were the partial promotion of muscle synthesis by upregulation of the Akt-mTOR pathway and the stimulation of cellular metabolism by increasing AMPK phosphorylation, without affecting the muscle hypertrophic effect (Yi et al., 2022).

#### 5.7.7 Hepatoprotective effect

The effects of maca on liver tissue have been reported in multiple studies, primarily to evaluate the safety of its consumption or within secondary objectives. However, an *in vivo* study directly evaluated its hepatoprotective effect and its possible mechanisms in mice with CYP-induced hepatotoxicity, providing oral administration of polysaccharide MP, a compound isolated from maca A decrease in histopathological changes and in serum transaminase and hepatic MDA levels was observed, associated with an increase in the levels of hepatic antioxidant enzymes (SOD, GSH-Px, CAT) and enzymes related to energy metabolism (ATPase, Na^+^/K^+^/ATPase), reflecting their effect against lipid peroxidation and oxidative stress. The possible mechanisms involved were the metabolic regulation of glycerophospholipids, arachidonic acid, the pyrimidine pathway, the pentose phosphate pathway and amino acids. In addition, it was proposed that this mechanism was related to a mitochondrial signaling pathway (Keap1-Nrf2) (Fei et al., 2022a).

#### 5.7.8 Proangiogenic, antithrombotic and antiallergic effect

An *in vitro* study evaluated other possible effects of maca, including its proangiogenic, antithrombotic and antiallergic activity. Some tests were performed on endothelial cells (EPC, HUVEC), human blood tissue and a basophilic leukemia cell line (RBL-2H3) applying the ethanolic extract of maca and some of its fractions (rich in macamide, n-hexane, aqueous, and 75% methanol) and components (macapirrolins A, B, D and E). The macamide-rich fraction exhibited an effect similar to vascular endothelial growth factor (VEGF), increasing cell growth; whereas the ethanolic extract exhibited an antithrombotic effect by reducing thrombus area; finally, macapirrolin A exhibited the highest superoxide inhibition and histamine degranulation comparable to azelastine (Purnomo et al., 2021).

## 6 Clinical studies

Among 22 clinical studies on maca, most focused on effects on sexual health (18), summarized in Table 1, and only a few evaluated effects on physical performance (2), metabolic syndrome (1), or overall health (1).

**TABLE 1:** Sexual health clinical effects studied for *Lepidium meyenii*.

Male sexual health			
Objetive	Study short description	Results	Ref.
Evaluate the effect on seminal parameters in male infertility	Pilot clinical trial, randomized, double-blind, controlled, parallel, with 65 males aged 20 to 40 with mild asthenozoospermia and/or oligozoospermia who received 1,000 mg enteric-coated capsules of powdered maca orally for 12 weeks	Increase in sperm concentration. No differences were observed in seminal volume, sperm motility, and morphology	Alcalde and Rabasa (2020)
Assessing the effect on late-onset hypogonadism	Randomized, double-blind, controlled, multicenter clinical trial involving 80 eugonadal males over 40 years with symptoms of late-onset hypogonadism who received 833 mg gelatinized maca capsules orally for 12 weeks	Reduction in symptoms of androgen deficiency, erectile dysfunction, and lower urinary tract symptoms	Shin et al. (2023)
Determine the effect on semen analysis	Unspecified clinical trial involving nine healthy adult males aged 24 to 44, who received 500 mg gelatinized maca tablets orally for 4 months	Increase in seminal volume, count of mobile spermatozoa, and motility (grade a+b). No difference in hormonal levels (FSH, LH, PRL, T, E_2_)	Gonzales et al. (2001)
Determine the effect on sexual desire associated with mood or serum testosterone levels	Randomized, double-blind, controlled, parallel clinical trial involving 57 healthy males aged 21 to 56, who received 500 mg gelatinized maca tablets orally for 12 weeks	Increase in sexual desire, independent of changes in mood, and serum testosterone and E_2_ levels	Gonzales et al. (2002)
Assess the effect on serum levels of reproductive hormones in males	Randomized, double-blind, controlled, parallel clinical trial with 56 healthy males aged 21 to 56, who received 500 mg gelatinized maca tablets orally for 12 weeks	There were no significant differences in levels of LH, FSH, and PRL between groups. Additionally, there were no significant differences in levels of 17-OHP, T, and E_2_ between groups	Gonzales et al. (2003)
Assess the impact on seminal parameters and hormonal levels	Pilot clinical trial, randomized, double-blind, controlled, with 13 healthy males aged 20 to 40, who received 350 mg capsules of gelatinized yellow maca orally for 12 weeks	Non-significant improvement in seminal quality parameters observed. No differences were noted in evaluated hormonal levels: LH, FSH, E_2_, T, fT4, TSH	Melnikovova et al. (2015)
Assess the effect on subjective wellbeing, both sexual and non-sexual	Randomized, double-blind clinical trial involving 50 young adults with mild erectile dysfunction who received 1,200 mg tablets of powdered dehydrated maca orally for 12 weeks	Significant improvement in erectile function. Enhancement observed in psychological, physical, and social performance	Zenico et al. (2009)
Assess the effects on male infertility	Randomized, double-blind, controlled clinical trial, ITT analysis, involving 50 infertile males aged 28 to 52, who received 400 mg capsules of gelatinized yellow maca orally for 16 weeks	No significant differences were observed in seminal parameters and hormonal levels (LH, FSH, PRL, E2, T, fT)	Melnikovova et al. (2021)
Women’s sexual health			
Assess the impact on menopausal symptoms and hormonal profile	Pilot clinical trial, randomized, double-blind, controlled, crossover study involving 13 (Trial I) and eight (Trial II) postmenopausal women aged 45 to 62, who received 500 mg gelatinized maca capsules orally for up to 9 months	Increase in LH, E2, and PG levels, and a decrease in FSH levels. Reduction in the severity of menopausal symptoms	Meissner et al., 2005
Assess the effect on menopausal symptoms	Pilot clinical trial, randomized, double-blind, controlled, crossover study involving 18 perimenopausal women aged 41 to 50, who received 500 mg pre-gelatinized maca capsules orally for 4 months	Increase in FSH and E2 levels. Reduction in the severity of menopausal symptoms	Meissner et al. (2006c)
Assess the effect on levels of sex hormones and menopausal symptoms	Randomized, double-blind, controlled, multicenter clinical trial involving 88 (Trial I) and 66 (Trial II) postmenopausal women aged 45 to 58, who received 500 mg pre-gelatinized maca capsules orally for up to 4 months	Decrease in FSH levels and increase in E2. Reduction in the severity of menopausal symptoms	Meissner et al. (2006d)
Assess the effect on hormonal parameters and menopausal symptoms	Randomized, double-blind, controlled, crossover clinical trial involving 34 postmenopausal women aged 45 to 58, who received 500 mg pre-gelatinized maca capsules orally for 4 months	Increase in E_2_ and decrease in FSH, LH, T3, cortisol, and ACTH. Progressive reduction in the frequency and severity of menopausal symptoms	Meissner et al. (2006e)
Assess the effect on hormonal profile and menopausal symptoms	Randomized, double-blind, controlled, crossover clinical trial involving 14 symptomatic postmenopausal women aged 50 to 60, who received powdered maca orally for 12 weeks	There were no significant differences in levels of E2, FSH, LH, and sex hormone-binding globulin (SHBG). Reduction in menopausal symptoms in psychological, anxiety, depression, and sexual dysfunction dimensions	Brooks et al. (2008)
Assess the effect on menopausal symptoms	Pilot randomized, double-blind, controlled, single-center, crossover clinical trial involving 29 symptomatic postmenopausal women aged 46 to 59, who received powdered maca orally for 12 weeks	Reduction in menopausal symptoms in the psychological, anxiety, and depression dimensions	Stojanovska et al. (2015)

### 6.1 Men’s sexual health

The effects on sexual health and fertility of healthy males have been evaluated in four studies, which used oral administration of gelatinized maca in tablets (Gonzales et al., 2001; Gonzales et al., 2002; Gonzales et al., 2003) or capsules (Melnikovova et al., 2015) for 12 weeks (Gonzales et al., 2002; Gonzales et al., 2003; Melnikovova et al., 2015 to 4 months (Gonzales et al., 2001), with no adverse effects reported. A first study only compared the administration of two different doses of gelatinized maca (1.5 vs. 3 g/day) for 4 months and found no significant differences between their effects, which included seminal volume increase, total sperm count, motile sperm count and sperm motility (grade a + b). Regarding hormone levels, this same study did not show a significant modification in the levels of FSH, LH, prolactin (PRL), T and E_2_ (Gonzales et al., 2001).

A subsequent study, which added a placebo arm and had a treatment duration of 12 weeks also obtained similar results, not finding significant differences between the levels of LH, FSH, PRL, 17-hydroxyprogesterone (17-OH), T and E2 between the studied groups (Gonzales et al., 2003). A more recent study applying the administration of 1.75 g/day of gelatinized maca for 12 weeks, compared with a placebo arm, did not obtain significant improvements in seminal quality parameters (Melnikovova et al., 2015).

Another study determined the effect on sexual desire in healthy men by applying the administration of 1.5 and 3 g/day of gelatinized maca for 12 weeks, compared with a placebo arm. It was obtained that the administration of both doses presented an increase in sexual desire at 8 and 12 weeks of treatment, this being independent of changes in mood and serum levels of T and E2 (Gonzales et al., 2002).

The effects on sexual health and fertility of males with some underlying impairment, have been evaluated in four studies, including mild erectile dysfunction (Zenico et al., 2009), male infertility (Alcalde et al., 2020; Melnikovova et al., 2021), and late-presenting hypogonadism (Shin et al., 2023). The studies in question used oral administration of maca powder in tablets of 1,000 mg (Alcalde et al., 2020) and 1,200 mg (Zenico et al., 2009), and gelatinized maca in capsules of 400 mg (Melnikovova et al., 2021) and 833 mg (Shin et al., 2023); with daily doses between 2 g (Alcalde et al., 2020) and 2.4 g (Zenico et al., 2009) of maca powder, and 2.8 g (Melnikovova et al., 2021) and approximately 5 g (Shin et al., 2023) of gelatinized maca, for 12 (Zenico et al., 2009; Alcalde et al., 2020; Shin et al., 2023) to 16 weeks (Melnikovova et al., 2021).

Administration of 2.4 g/day of maca powder for 12 weeks in mild erectile dysfunction had positive effects on subjective sexual wellbeing, observing an increase in erectile function and psychological, physical and social performance, compared to placebo (Zenico et al., 2009). On the other hand, the use of 2 g/day of infertile males with mild asthenozoospermia and/or oligozoospermia reported an increase in sperm concentration without evidence of significant changes in seminal volume, sperm motility and morphology (Alcalde et al., 2020).

Effects on seminal parameters in infertile males with various combinations of oligospermia, asthenozoospermia, teratozoospermia, and azoospermia were reported in an intention-to-treat trial. After receiving treatment with 2.8 g/day of gelatinized maca for 16 weeks, there was no change in seminal parameters or levels of LH, FSH, PRL, E2, T and thyroid hormone (fT) compared to placebo (Melnikovova et al., 2021).

Recent research proposed evaluating the effect in eugonadal males over 40 years of age with symptoms of late-presenting hypogonadism, or also known as andropause. This study applied the oral administration of approximately 5 g/day of gelatinized maca for 12 weeks, presenting a significant decrease in symptoms of androgen deficiency, erectile dysfunction and lower urinary tract, compared to baseline and placebo. As in the studies already mentioned, there were also no significant differences between T and fT levels, and other parameters such as PSA (prostate-specific antigen), high-density lipoprotein-cholesterol (HDL-C), low-density lipoprotein-cholesterol (LDL-C) and triglicerides (Shin et al., 2023).

### 6.2 Women’s sexual health

The effects on sexual health of healthy women have been evaluated using oral administration of capsules of an herbal supplement containing maca glucosinolates and phytosterols, at different daily doses (1,650 and 3,300 mg) for 3 months. Neither moderate or severe adverse effects, nor immediate or delayed hypersensitivity reactions were reported. Likewise, there were no significant differences in anthropometric measures, hematological, renal, hepatic, lipid profile and sex hormone evaluations. However, an increase in basal glucose and a decrease in uric acid were reported at 1,650 mg/day together with a decrease in respiratory rate and an increase in basal temperature and SBP with 3,300 mg/day, compared to placebo (Villar-López et al., 2021).

The effects on sexual health and fertility of menopausal women have been evaluated in seven studies (Meissner et al., 2005; Meissner et al., 2006a; Meissner et al., 2006b; Meissner et al., 2015; Brooks et al., 2008; Stojanovska et al., 2015), which employed oral administration of gelatinized/pregelatinized maca capsules (Meissner et al., 2005; Meissner et al., 2006a; Meissner et al., 2006b; Meissner et al., 2015) and maca powder (Brooks et al., 2008; Stojanovska et al., 2015) for 12 weeks (Meissner et al., 2005; Meissner et al., 2006b; Brooks et al., 2008; Stojanovska et al., 2015), 4 months (Meissner et al., 2006a; Meissner et al., 2006b; Meissner et al., 2015), and up to 9 months (Meissner et al., 2005a).

Studies focused on the effects on menopause were crossover clinical trials, in which hormone levels and symptom severity were measured. Within hormone levels, there were conflicting results regarding FSH and E2 levels. Daily administration of 2 g of gelatinized/pregelatinized maca showed a decrease in FSH along with an increase in E2 (Meissner et al., 2005; Meissner et al., 2006b; Meissner et al., 2015), but an increase in both hormones has also been reported (Meissner et al., 2006a). On the other hand, the daily administration of 3.5 g of maca powder did not show significant differences in FSH or E2 levels (Brooks et al., 2008).

Regarding the severity of menopausal symptoms, all reviewed studies agree that the administration of gelatinized/pregelatinized maca and maca powder decreased the severity of these symptoms according to the Greene climacteric scale (GCS) (Meissner et al., 2005; Meissner et al., 2006b; Meissner et al., 2015; Brooks et al., 2008; Stojanovska et al., 2015), and the Kupperman menopausal index (KMI) (Meissner et al., 2006a; Meissner et al., 2006b; Meissner et al., 2015). A decrease in menopausal symptoms of the psychological dimensions, anxiety, depression (Brooks et al., 2008; Stojanovska et al., 2015) and sexual dysfunction (Brooks et al., 2008) was reported according to the GCS; and a decrease in hot flashes, disrupted sleep pattern, depression and excessive sweating according to the KMI (Meissner et al., 2006a). Another study also analyzed the effect of maca powder administration on other aspects of life, where there was evidence of improvement in general health, mental health, social functioning and mental components according to the SF-36 version 2 health questionnaire (SF-36v2), and on the depression, somatic, anxiety and sleep scales according to the women’s health questionnaire (WHQ) (Stojanovska et al., 2015).

One study focused on the effects of maca on female fertility, specifically in women of reproductive age with disorders of the hypothalamic-pituitary-ovarian axis. The study compared the daily administration of a proprietary blend containing maca extract and a nutritional complex. No significant differences in the incidence of pregnancies were reported between the two groups (Antoine Edu et al., 2019). Other outcomes such as possible effects on ovulation and polycystic ovary syndrome are reported together, without establishing a comparison between study groups.

### 6.3 Sexual health and antidepressants

Two studies evaluated the effects of maca in people with sexual dysfunction from treatment with antidepressant drugs. One of them used oral administration of maca capsules for 12 weeks in patients using selective serotonin reuptake inhibitors (SSRIs). Different daily doses (1.5 and 3 g) were evaluated, obtaining an improvement in global sexual function according to the Arizona Sexual Experience Scale (ASEX) compared to placebo. However, this improvement was not evident when applying the Massachusetts General Hospital Sexual Functioning Questionnaire (MGH-SFQ) or in libido according to ASEX and MGH-SFQ. Similarly, no significant differences were observed in attempts at sexual activity, satisfaction and orgasms or in symptoms of depression and anxiety (Dording et al., 2008).

A subsequent study evaluated the effect of maca on antidepressant-induced female sexual dysfunction (AISD) by administering a daily dose of 3 g of maca capsules for 12 weeks. The results, based on a modified intention-to-treat analysis (mITT), showed an improvement in the scores obtained in the ASEX and in the orgasm dimension of MGH-SFQ in postmenopausal women (Dording et al., 2008).

### 6.4 Physical performance

The effect of maca on physical performance was evaluated in two small studies with male professional athletes (Ronceros et al., 2005; Stone et al., 2009). The first applied the oral administration of micropulverized fresh maca capsules for 60 days in doses of 1.5 g/day. This study reported an increase in physical performance by 10.3%, represented by the increase in maximum speed and maximum oxygen consumption (VMO2) compared to baseline. In addition, no significant changes were reported in liver (OGT, TGP) and renal (Cr) markers (Ronceros et al., 2005).

A second study, the product of a crossover clinical trial, applied the oral administration of maca extract capsules for 2 weeks at doses of 2 g/day. An improvement in endurance exercise performance was reported when showing a decrease in the time to complete 40 km. In addition, and an increase in sexual desire was also observed according to the Sexual Desire Inventory (SDI) (Stone et al., 2009).

### 6.5 Metabolic syndrome

The safety of maca in patients with metabolic syndrome was evaluated using oral administration of dehydrated maca capsules alone or associated with 100 mg of silymarin at doses of 600 mg/day and 600 + 200 mg/day for 90 days, respectively. An increased level of liver transaminase (AST) and DBP was observed in the maca-alone group; while in the group of associated maca and silymarin, these effects were not evidenced (Valentová et al., 2008).

### 6.6 General health

Finally, a study evaluated maca consumption in healthy adults from two geographically distinct areas: altitude (4,340 masl) and sea level (150 masl). Oral administration of atomized extracts of black and red maca was used for 12 weeks, at doses of 3 g/day. An increase in the perception of sexual desire was observed in 50% of both groups, with a possible placebo effect compared to black maca. There was also an improvement in health-related quality of life (HRQL) compared to placebo, as well as a decrease in SBP in the groups treated with black maca compared to the other groups. In the group of participants living at altitude, a decrease in Hb levels and chronic mountain sickness was reported in those treated with black and red maca, respectively. On the other hand, a possible placebo effect on mood perception and greater acceptability of maca was reported. In the group of participants living at sea level, an increased perception of mood was reported compared to placebo. No serious adverse effects were reported in any study group (Gonzales-Arimborgo et al., 2016).

## 7 Discussion

In recent years, maca has gained attention in the scientific community for its potential health benefits. Researchers have explored its pharmacological properties and have identified bioactive compounds that may contribute to its effects. We have reviewed studies looking carefully into findings details, consistency and reliability across different preclinical models, their potential therapeutic effects *in vitro*, as well as animal models and human clinical trials.

The precise molecular mechanisms underlying the bioactivities of maca are still under investigation, but generally we can attribute maca its well-known adaptogenic and neuroprotective to its rich phytochemical composition. Its physiological properties arise from the synergistic combination of different compounds, providing a unique profile not attributable to a single molecule. Recent evidence suggests that maca’s effects on the hypothalamus-pituitary adrenals (HPA) axis involve a probable synergistic mechanism, wtih macamides as the mediator through the serotoninergic pathway via CBs receptors, with multiple physiological consequences.

Maca demonstrates neuroprotective effects in various *in vitro* and *in vivo* models, with different maca ingredients, formulations and compounds showing efficacy in preserving neuronal cell viability and reducing neurotoxicity markers. This effect can be explained in part because of the various mechanisms involved including interactions with proteins in the NF-κB pathway, with cannabinoid receptors, and modulation of apoptotic proteins. Maca exhibits antioxidant, anti-inflammatory and analgesic effects in both *in vitro* and *in vivo* studies, demonstrating the attenuation of inflammation in models of hepatitis, pulmonary fibrosis, and benign prostatic hyperplasia.

It is important to highlight for secondary metabolites the information regarding doses for therapeutic benefits and potential adverse effects associated with higher doses. Toxicology studies performed with different types of ingredients, including maca powder, gelatinized maca powder and different types of extracts as well as with isolated macamides, demonstrated no serious adverse health effects, which allowed to complete the admission evaluation process of maca’s novel ingredients towards USP-NF monograph compendia The actual evidence indicate that no fatal adverse effects have been reported for maca, reinforcing its traditional use as a food source and medicinal plant.

At preclinical stage, studies, especially *in vitro*, have consistently shown low toxicity levels at various concentrations. Numerous studies indicate low acute oral toxicity in animals, with LD50 values well above typical consumption levels. There is also limited evidence of acute neurological and liver toxicity when evaluating different extracts, further supporting its safety, which is later demonstrated also on the clinical setting, encouraging its use for potential therapeutic applications.

Human studies involving oral administration of maca powder or capsules over several weeks generally reported good tolerance, with no serious adverse effects observed at varying doses. Some studies exemplifying this, including those involving daily consumption of fresh micro-pulverized maca for an extended period, did not find evidence of liver or renal toxicity for long term consumption. In addition, solid evidence arises from a large observational study in the native region of maca consumption, reporting its safety, with no significant differences in clinical parameters, liver and kidney function, and lipid profile between regular and non-habitual users.

While most studies report good tolerance towards maca, there are some exceptions to consider, since transient adverse events such as gastrointestinal upset, headache, and irritability were reported and other compromising effects such as vaginal bleeding and a manic episode in individuals were also found. The occurrence of adverse effects appears to vary among individuals, suggesting that maca’s impact may be influenced by factors such as individual physiology, dosage, and duration of use.

The array of clinical studies on maca provides valuable insights into its potential effects on various aspects of human health. Primarily, the studies appear to be oriented towards evaluating maca’s impact on sexual health, with a focus on men’s and women’s reproductive systems, demonstrating maca’s potential contributions to improved male and female fertility by enhancing sperm quality and testosterone levels, underscoring its potential as a valuable dietary supplement. Additionally, a few studies explore its effects on physical performance and ability to combat fatigue, enhance energy metabolism, potentially alleviate symptoms of metabolic syndrome, along with its positive influence on gastrointestinal function, and overall health.

## 8 Conclusion

While more research is needed to fully understand its mechanisms and potential therapeutic applications, is remarkable to spot the alignment of the ethnopharmacological studies with many of the traditional uses attributed to Maca, as being an adaptogenic plant with multiple and diverse pharmacological implications, further solidifying its role in promoting overall wellbeing. Ongoing research has unveiled maca’s promising attributes in various areas, including cardioprotection, antihypertensive properties, photoprotection, anabolic effects, hepatoprotection, and even proangiogenic, antithrombotic, and antiallergic activities. Maca exhibits multifaceted potential for health enhancement, with a particular emphasis on its neuroprotective effects and significant contributions to sexual health, marking them as the most promising bioactivities warranting further research.

Considering the translational potential of these preclinical and clinical findings, along with its practical implications, some challenges should be taken in consideration in the clinical settings, as further studies are needed to more comprehensively underscore maca’s potential health benefits. The preclinical and clinical studies on maca provide valuable insights into its potential health benefits. However, the variability in study designs, dosages, and treatment durations opens a window for standardized protocols to enhance the reliability and comparability of findings. Future research should address these methodological challenges to establish a more comprehensive understanding of maca’s impact on human health. In conclusion, maca stands out as a remarkable natural ingredient with a broad spectrum of health benefits. Exploring its effects on applications including neuroprotection and overall health may uncover additional dimensions of its health-enhancing capabilities.
