# Supplementary material for: Exploring the chemical and pharmacological variability of Lepidium meyenii: a comprehensive review of the effects of maca
Source: Front Pharmacol. 2024 Feb 19;15:1360422. doi: 10.3389/fphar.2024.1360422 (PMC10910417; doi:10.3389/fphar.2024.1360422)
Supplement: Supplementary file 1 [file DataSheet1.PDF]

Supplementary Material

1 Supplementary Figures and Tables

Table S1 Preclinical and Clinical Studies Reporting Maca Toxicology

Preclinical Studies Reporting Maca Toxicology

Objective	Presentation/ Formulation	Dosage	Treatment time	Toxicology	Reference
To evaluate effects in pre-implantation embryos of mice	Lyophilized aqueous extract of yellow maca	1g/kg/day intraperitoneal	Four days	No alteration in the normal development of embryos was evidenced	D'Arrigo, 2004
To assess short- and long-term physiological effects in rats	Pregelatinized Maca	7.5g/kg/day PO	28 days (short term)<br><br>90 days (long term)	There were no histopathological changes in internal organs (liver, spleen, pancreas) or hematological changes; suggesting an LD50 >7.5g/kg	Meissner, 2006a
To evaluate *in vitro* effects of high doses of maca on hepatocytes of male rats	Methanolic and aqueous extracts of dried maca hypocotyls	≤10mg/ml	Single dose	There was no significant influence on cell morphology and viability or liver enzyme levels (AST, LDH) after 72 hours of incubation	Valentová, 2006
To evaluate acute toxicity in rats and mice, and hormonal and cognitive-	Pregelatinized Maca	15g/kg/day PO (mice)	Single dose	There were no changes in behavior and weight, nor histopathological alterations in the liver, spleen, pancreas, testicles	Meissner, 2006b

behavioral regulatory effects in oophorectomized rats		5g/kg/day PO (rats)		and ovaries; suggesting an LD50 >15g/kg in mice and >5g/kg in rats	
To evaluate chronic effects on reproductive parameters and DNA quantification of adult male rats	Lyophilized aqueous extracts of red, yellow and black maca	1g/kg/day PO	84 days	No histological liver changes were evidenced	Gasco, 2007
To assess effects on persistent pain in rats and mice	Aqueous extract of maca root	10g/kg PO	Single dose	No motor or neurological alterations were evidenced	Tenci, 2017
To evaluate anti-fatigue effect in mice	Dried roots of yellow maca powder	1.2g/kg/day PO	30 days	Significantly increased serum urea nitrogen (BUN) levels	Li, 2017
To evaluate metabolic effect *in vitro* in adipocytes	Ethanolic extract of red maca hypocotyls (ME)	≤500µg/ml	Single dose	Preservation of cell viability after 48 hours of incubation	Chen, 2019
To evaluate safety and protective effects against neurotoxicity in rats and mice	Ethanolic extract of dried maca	1g/kg/day PO<br><br>2g/kg/day PO	45 days (Subacute toxicity test)<br><br>Single dose (Acute toxicity test)	There were no changes in hematological and biochemical parameters, as well as weight and histological structure of organs.<br><br>No changes in histological structure of organs were evidenced, suggesting an LD50 >2g/kg	Yu, 2020a

To assess in vitro *anti-fatigue effect* on skeletal muscle cells	Lyophilized extract of black maca (ME)	≤1 mg/ml	Single dose	No cytotoxicity was evidenced, maintaining the percentage of cell viability after 24 hours of incubation	Zhu, 2021
To assess effects on neonatal hypoxic-ischaemic brain injury in newborn mice	Synthesized compound of maca (Macamide B)	60mg/kg intraperitoneal	Single dose	There were no morphological or histopathological changes in liver and kidney	Yang, 2022

Clinical Studies Reporting Maca Toxicology

**Objective and sample**	**Presentation/Formulation**	**Dosage**	**Treatment time**	**Toxicology**	**Reference**
To evaluate effects on physical performance of athletes at altitude	Capsules with 500mg of fresh micropulverized maca	1.5g/day PO	60 days	There were no changes in liver (OGT, TGP) and renal (Cr) markers	Ronceros, 2005
To assess effects on sexual dysfunction induced by selective serotonin reuptake inhibitors (SSRIs) in people diagnosed with depression	Capsules with 500mg of maca powder	3g/day VO	12 weeks	There was good tolerance overall, but there were reports of transient adverse events: gastrointestinal upset, headache, irritability, panic attack, pollakiuria, blurred vision, sleep disruption, increased sweating, increased sleep,	Dording, 2008

				increased menstrual flow and exacerbation of fibromyalgia	
Evaluate safety in patients with metabolic syndrome	Capsules with 300mg of maca powder	0.6g/kg/day PO	90 days	Moderate increase in AST and diastolic BP level	Valentová, 2008
To evaluate food and nutritional security in people with and without traditional maca consumption, in an observational way	Dried maca hypocotyls	>260g/day VO	Variable	There were no differences in liver and kidney markers	Gonzales, 2010
To assess acceptability, safety and efficacy in apparently healthy adults living at altitude and sea level	Spray-dried maca extracts	3g/day VO	12 weeks	No serious adverse effects were reported	Gonzales, 2016
Effects on seminal quality of patients with mild asthenozoospermia and/or oligozoospermia	Enterosolvent gelatin capsules with 1000 mg maca powder	2g/day PO	12 weeks	No adverse effects were reported	Alcalde, 2020
To assess safety and tolerability in premenopausal adult women	Capsules with 550mg oral herbal supplement with glucosinolates, b-sitosterol and citrus flavonoids (Warmi®)	3.3g/day PO	3 months	No moderate or severe adverse effects or immediate or delayed hypersensitivity reactions were reported.<br><br>There was no evidence of changes in hematological, renal or hepatic parameters	Villar, 2021

To assess efficacy and safety in men with late-presenting hypogonadism	Capsules with 833mg of gelatinized maca powder	≈5g/day PO	30 days	Gastrointestinal disturbances were reported as mild symptoms that did not affect study participation.<br><br>No serious side effects were reported.<br><br>There were no significant changes in laboratory tests or vital signs.	Shin, 2023
